# USP5 regulates purine metabolism and represents a therapeutic target in esophageal cancer

**DOI:** 10.1038/s41419-026-08683-4

**Published:** 2026-04-01

**Authors:** Kexin Zhao, Lei Zhang, Mingyang Yan, Shaobo Fang, Zhenwei Wang, Yanming Song, Meng Liu, Chengjuan Zhang, Yang Shao, Xinyang Jia, Qinxin Guo, Manman Guo, Junyuan Yin, Guoguo Jin, Zigang Dong

**Affiliations:** 1 China-US (Henan) Hormel Cancer Institute, No. 127, Dongming Road, Jinshui District, Zhengzhou, China; 2 Department of Pathophysiology, School of Basic Medical Sciences, Zhengzhou University, Zhengzhou, China; 3 Department of Radiology, Zhengzhou University People’s Hospital & Henan Provincial People’s Hospital, Zhengzhou, China; 4 Henan Key Laboratory of Chronic Disease Management, Fuwai Central China Cardiovascular Hospital, Zhengzhou, China; 5 Department of Pathology, Affiliated Cancer Hospital of Zhengzhou University & Henan Cancer Hospital, Zhengzhou, China; 6 State Key Laboratory of Metabolic Dysregulation & Prevention and Treatment of Esophageal Cancer; School of Basic Medical Sciences, Zhengzhou University, Zhengzhou, China; 7 Tianjian Laboratory of Advanced Biomedical Sciences, Institute of Advanced Biomedical Sciences, Zhengzhou University, Zhengzhou, China; 8 Central China Subcenter of National Center for Cardiovascular Diseases, Henan Cardiovascular Disease Center, Fuwai Central-China Cardiovascular Hospital, Central China Fuwai Hospital of Zhengzhou University, Zhengzhou, China

**Keywords:** Cancer microenvironment, Biomarkers

## Abstract

Ubiquitination is a critical regulator of cancer development, yet the role of deubiquitinases in purine metabolism remains largely unexplored. In this study, untargeted metabolomic analysis revealed a significant upregulation of purine metabolism in esophageal cancer (ESCC). Database analysis further identified a strong positive correlation between the deubiquitinase USP5 and purine metabolism. Functional assays demonstrated that USP5 knockdown suppressed cell proliferation in vitro and inhibited tumor growth in vivo, accompanied by a reduction in purine metabolism. Mechanistically, USP5 directly interacts with inosine monophosphate dehydrogenase 2 (IMPDH2), the rate-limiting enzyme in de novo guanine nucleotide biosynthesis, and removes K48-linked polyubiquitin chains at lysine 489. This deubiquitination event stabilizes IMPDH2, prevents its proteasomal degradation, and promotes guanine nucleotide synthesis. Guanine supplementation enhanced ESCC cell proliferation, whereas dietary purine restriction suppressed tumor progression in vivo. Furthermore, we identified mebendazole, an FDA-approved anthelmintic, as a pharmacological inhibitor of USP5. Combination treatment with mebendazole and oxaliplatin significantly enhanced chemosensitivity in ESCC cells. Collectively, these findings establish the USP5-IMPDH2-guanine axis as a critical driver of ESCC progression and highlight its potential as a promising therapeutic target for ESCC.

## Introduction

Esophageal squamous cell carcinoma (ESCC) is the sixth leading cause of cancer-related death globally, with high incidence rates in East Asia, Eastern Africa, and South America [1]. In China, ESCC constitutes over 90% of esophageal cancer cases, predominantly affecting elderly males in rural regions [2]. Despite advances in surgery, chemoradiotherapy, and targeted therapies, the 5-year survival rate remains below 20%, largely due to its aggressive biology, early metastasis, and lack of effective early detection [3, 4]. Among the hallmarks of cancer, metabolic reprogramming plays a central role in sustaining tumor growth and survival [5]. ESCC cells, like many other malignancies, preferentially adopt aerobic glycolysis (the Warburg effect) and alter glutamine, lipid, and redox metabolism to support anabolic demands and resist oxidative stress [6, 7]. These metabolic shifts are orchestrated by key oncogenic pathways, including MYC, PI3K-AKT-mTOR, and HIF1α. Importantly, metabolic plasticity also contributes to immune evasion and therapeutic resistance, underscoring the potential of targeting tumor metabolism as a complementary approach to current treatments [8, 9]. ESCC remains a deadly malignancy with urgent unmet needs in early detection, effective biomarkers, and novel therapeutic strategies to overcome poor prognosis and treatment resistance.

The ubiquitin-proteasome system (UPS) is one of the major protein degradation pathways in cells and plays a critical role in maintaining protein homeostasis, regulating the cell cycle, signal transduction, DNA repair, and immune responses [10, 11]. In addition, deubiquitinases (DUBs) can hydrolyze ubiquitin chains to regulate protein stability and recycle ubiquitin [12]. Dysregulation of the UPS is closely associated with various diseases, including cancer, neurodegenerative disorders, and autoimmune diseases [13]. Within the large family of deubiquitinases, ubiquitin-specific protease 5 (USP5), is unique in its ability to cleave unanchored polyubiquitin chains and recycle free ubiquitin monomers [14]. USP5 has been implicated in various physiological and pathological processes, including DNA damage response, cellular stress signaling, and inflammatory regulation [15]. Notably, emerging evidence indicates that USP5 was dysregulated in several types of cancer and contributes to tumorigenesis by stabilizing oncogenic proteins [16]. Previous studies have demonstrated that USP5 exerts oncogenic functions primarily through its deubiquitinating activity by stabilizing tumor-promoting substrates, including STAT3, thereby enhancing tumor angiogenesis and cancer progression [17, 18]. However, the role of USP5 in metabolic regulation, particularly in purine metabolism, has not yet been reported.

Purine metabolism plays a vital role in nucleotide biosynthesis, energy transfer, and cellular signaling [19]. Through a combination of untargeted metabolomics and ¹³C -labeled metabolic flux analysis, we identified a significant dysregulation of purine metabolism in ESCC. We systematically screened all known enzymes involved in the de novo purine biosynthetic pathway [20]. Among these, inosine monophosphate dehydrogenase 2 (IMPDH2) emerged as a key enzyme [21]. IMPDH2 catalyzes the rate-limiting step in guanine nucleotide synthesis, converting IMP to xanthosine monophosphate (XMP), which is further converted into GMP and eventually into Guanine [22, 23]. Elevated IMPDH2 expression has been previously implicated in a variety of malignancies, including glioblastoma, leukemia, colorectal cancer, and prostate cancer, and was often associated with aggressive tumor behavior and poor prognosis [24, 25]. Furthermore, recent studies have linked IMPDH2 expression with oncogenic signaling pathways such as MYC and mTOR, positioning it as a downstream effector of oncogenic metabolic reprogramming [26]. However, its regulatory mechanisms and functional consequences in ESCC have not been thoroughly characterized.

Herein, we identified that, USP5 stabilizes IMPDH2 by removing K48-linked polyubiquitin chains, thereby enhancing guanine nucleotide biosynthesis and promoting tumor proliferation. USP5 knockdown induced IMPDH2 degradation, leading to decreased guanine production, suppressed tumor cell proliferation, and impaired tumor growth in vitro and in vivo. we demonstrated that Mebendazole, an FDA-approved anthelmintic drug, acts as a pharmacological inhibitor of USP5, destabilizing IMPDH2 and reducing guanine nucleotide pool. We further found that the combination of Mebendazole with Oxaliplatin significantly enhanced the antitumor efficacy of Oxaliplatins. In a summary, our findings highlight the USP5–IMPDH2–guanine axis as a critical metabolic pathway in ESCC and propose USP5 inhibition as a promising therapeutic strategy to suppress tumor progression and overcome chemoresistance.

## Materials and methods

### Cell culture

The KYSE30 (CVCL_1351), KYSE450 (CVCL_1353), KYSE150 (CVCL_1348), KYSE510 (CVCL_1354), KYSE70 (CVCL_1356) and KYSE410 (CVCL_1352) ESCC cell lines were obtained from the Cell Bank of the Chinese Academy of Sciences (Shanghai, China). The immortalized normal esophageal epithelial cell line SHEE (CVCL_4M96) was generously provided by Dr. Enmin Li (Shantou University, Guangdong, China). Cell line authentication was performed by Sangon Biotech (Shanghai) in July 2022 using short tandem repeat (STR) analysis, and all cell lines matched their reference profiles. Prior to experimental use, mycoplasma contamination was assessed using a commercial mycoplasma detection kit, and all cell lines were verified to be mycoplasma-free. KYSE30, KYSE450, KYSE150, KYSE510, KYSE410, KYSE70 cells were maintained in RPIM- 1640 and HEK293T cells were maintained in Dulbecco’s modified Eagle’s medium (DMEM) and at 37°C under 5% CO_2_ supplemented with 10% fetal bovine serum (FBS) and 1% penicillin–streptomycin. All cells were obtained from the Bank of Type Culture Collection of Chinese Academy of Sciences, recently authen ticated by fingerprinting of short tandem repeats, and tested negative for mycoplasma. For serine and glycine depletion, cells were cultured in serine- and glycine-free RPMI 1640 medium (US Biological Life Sciences, D980003) supplemented with 10% dialyzed FBS.

### Reagents and antibodies

Primary antibodies used in this study included anti-USP5 (Proteintech, 66213-1-Ig), anti-IMPDH2 (Proteintech, 67663-1-Ig), anti-ubiquitin (Proteintech, 10201-2-AP), anti-β-actin (Santa Cruz Biotechnology, sc-47778), anti-GAPDH (ZSGB-BIO, TA-08), anti-HNRNPC (Proteintech, 11760-1-AP), anti-G3BP1 (Proteintech, 13057-2-AP), anti-PPP1R10 (Proteintech, 24450-1-AP), anti-CNBP (Proteintech, 14717-1-AP), anti-DRG1 (Proteintech, 13190-1-AP), anti-HNRNPA1 (Proteintech, 11176-1-AP), anti-HNRNPA0 (Proteintech, 10848-1-AP), anti-SAFB (Proteintech, 21857-1-AP), anti-PTBP1 (Proteintech, 12582-1-AP), and anti-FBL (Proteintech, 16021-1-AP).

### Western blot

The collected cells were lysed on ice with RIPA buffer for an hour, and then centrifuged at 4 °C at 12000 rpm for 15 min in a high-speed centrifuge. The supernatant after centrifugation was taken and protein quantification was performed using a BCA Quantification Kit (Solarbio). The prepared protein sample was then boiled in a metal water bath at 95 °C for 10 min and then separated by SDS-PAGE. Separated proteins were then transferred to polyvinylidene difluoride membranes. After blocking with 5% defatted milk powder (diluted in TBST) for 90 min at room temperature, each membrane was then incubated with one of the following antibodies (1:1000):USP5, IMPDH2, SYVN1, GAPDH, p-AKT, AKT, p-GSK-3β, GSK-3β, p-mTor, mTor, myc-tag, FLAG-tag, HA-tag, Ubiquitin, β-actin with shaking at 4 °C overnight.The next day, after recovering the first antibody, wash three times with TBST for 10 min each time. Then apply the secondary antibody (Mouse or Rabbit) at room temperature for two hours, wash it three times with TBST, each time for ten minutes, and then expose it with an exposure machine.

### MTT assay

Inoculate cells at a density of 2000 cells per well into a 96 well plate and incubate overnight at 37 °C. Dilute each drug (Mebendazole) with different concentrations in culture medium, add to a 96 well plate, and incubate at 37 °C for 24 h. Dissolve MTT (Solarbio) powder at a concentration of 5 mg/ml in sterilized PBS and filter. Add 20 μL of MTT to the tablet and incubate at 37 °C for 2 h. Then extract the culture medium from the plate and use 100 μL Substitute with DMSO. Then gently stir the plate and measure the absorbance of each well at 490 and 570 nm using an enzyme-linked immunosorbent assay (ELISA) reader.

### Soft-agar colony formation assay

Anchorage-independent growth was evaluated using a soft-agar colony formation assay. Briefly, a base layer of normal growth medium containing 0.5% agar was first added to each well of a 6-well plate and allowed to solidify. ESCC cells were then suspended in normal growth medium containing 0.33% agar and seeded onto the base layer in the presence of the indicated concentrations of indomethacin. The plates were incubated at 37 °C for 10–14 days. Colonies were subsequently imaged using an inverted microscope and quantified using Image-Pro Plus software.

### Foci formation assay

KYSE30, KYSE450, KYSE150, and KYSE510 ESCC cells were seeded into 6-well plates at an appropriate density and cultured under standard conditions (37 °C, 5% CO₂) for 10–14 days to allow colony formation. At the end of the incubation period, the culture medium was carefully removed, and the cells were fixed and stained with 5% crystal violet solution for 10 min at room temperature. Excess stain was removed by gently rinsing the wells with distilled water. Plates were then air-dried, photographed, and the number of colonies containing more than 50 cells was counted manually or using ImageJ software.

### Co-immunoprecipitation

Cells were lysed with RIPA buffer for an hour at 4 °C. A.External IP: Configure the system with NET-NW buffer (600 μL), tag beads(30 μL), and lysed protein (600–800 mg). B.Endogenous IP: The cell lysates (600–800 mg) were shaked with indicated antibody (1 μL) and protein A/G beads (60 μL) at 4 °C overnight. The next day, the beads were washed five times with NET-NW buffer and boiled for 10 min in 1× SB buffer. The eluted protein was subjected to Western blot.

### Immunofluorescence staining and confocal analysis

KYSE cells were co-transfected with Flag-USP5 and MYC-IMPDH2 for 48 h. The culture medium on the upper layer of the 12-well plate was removed and washed twice in 1× PBS for 5 min. PBS was added slowly along the wall of the plate to avoid washing away the cells. One milliliter of 4% paraformaldehyde was added to each well with slow shaking at room temperature for 20 min. After washing three times, the 0.1% Triton X-100 was added to each well for 15 min, followed by three washes with 1× PBS. Cells were incubated with primary antibody overnight in 1% TBST, followed by incubation with Alexa Fluor 568 goat anti mouse secondary Ab, Alexa Fluor 488 secondary antibody goat anti mouse secondary Ab. Nuclei were counterstained with anti-fade fluorescence mounting medium with DAPI.

### qRT-PCR

Total RNA was extracted from cells, tissue and isolated. cDNA was generated with the PrimeScript RT kit. TB Green Premix Ex Taq™ II measured relative cDNA abundance, and β-actin or GAPDH as an internal reference gene for target genes. The relative expression of target genes was calculated through the 2-ΔΔCT (ΔCT = CTtarget − CTcontrol) method and normalized to the relative expression tested in control cells (set to 1.0). For more than two gene analysis between two groups, we used 2-ΔCT (ΔCT = ΔCTtumour − ΔCT*β*-actin) method to calculate the gene expression level (defined as multiple changes).

### Immunohistochemistry

Immunohistochemistry (IHC) was performed to detect the expression of USP5 and IMPDH2. The ESCC tissue microarray used for IHC analysis was obtained from Shanghai Outdo Biotech Company, China (HEsoS180Su10). Primary antibodies included rabbit monoclonal antibodies against IMPDH2 and USP5, and a murine monoclonal antibody against Ki67 (1:400; GB121141; Servicebio Technology). Briefly, 4-µm-thick tissue sections were cut, deparaffinized in xylene, rehydrated through graded ethanol, and subjected to heat-induced antigen retrieval. Secondary antibodies were purchased from Santa Cruz Biotechnology. Protein expression was quantified as the percentage of positively stained tumor cells (range, 0–100%).

### Infect

Add 2 mL of virus, 7 mL of 1640 medium containing 10% FBS, and 9 μL of infection reagent polybrene to a culture dish with a cell density of 50%. After waiting for 24 h, the virus will fully infect the cells. Replace the culture medium with a medium containing puromycin to screen whether the virus has fully infected the cells. Cultivate in a 37 °C incubator for 48 hours to obtain cells successfully infected by the virus.

### Transfection

According to the cell density, passaging cells (with a cell density of about 70% on the second day), take out Opti MEM medium and DMEM medium containing FBS. Remove the target plasmid and packaging plasmids PSPAX2 and PMD2G. Drop 500uL Opti MEM into an EP tube, then add corresponding volumes of target plasmid, packaging plasmid PSPAX2, and PMD2G. Take another EP tube, add 500 μL Opti MEM, and then add the corresponding volume of Lipo2000 (1 μg plasmid added to 3 μL). Mix the liquids in two EP tubes and wait for 17 min. Replace the culture medium of Lenti-293 cells with DMEM supplemented only with FBS. After 17 min, the plasmid was added to the cells and cultured in a CO_2_ cell culture incubator. After waiting for 4–6 hours, switch to normal culture medium, and after 48 h, collect the cells that have been successfully transfected or the virus solution released by lenti-293T.

### Cell-derived xenograft

This study was approved by the Ethics Committee of Zhengzhou University (Zhengzhou, Henan, China). All animal procedures were performed in accordance with institutional guidelines and protocols approved by the Institutional Animal Care and Use Committee. Female BALB/c nude mice (6–8 weeks old), Vital River Laboratories, Beijing, China) were randomly assigned to experimental groups. Cells infected with the indicated lentiviruses were subcutaneously injected into the right flank of each mouse. Tumor volume was measured every two days using a vernier caliper and calculated using the formula *V* = length × width × height × 0.52. Mice were euthanized when tumor volume reached 1000 mm³, and tumors were subsequently harvested for analysis. No samples or animals were excluded from the analysis, and no pre-established inclusion or exclusion criteria were applied. No blinding was performed.

### Patient-derived xenograft

Animal studies were approved by the Ethics Committee of Zhengzhou University (Zhengzhou, Henan, China) and the Ethics Committee of the China–US (Henan) Hormel Cancer Institute. Female SCID immunodeficient mice (8 weeks old) were used for patient-derived xenograft experiments. Written informed consent was obtained from patients for the generation of PDX tumors. Tumor tissues were transplanted subcutaneously into mice, and experiments were initiated seven days after transplantation (designated as day 1). Tumor volume and body weight were measured twice weekly. Tumor volume was calculated using the formula length × width × height × 0.52. Mice were euthanized when tumor volumes reached approximately 1 cm³, after which tumors were excised, weighed, and photographed. Mice were randomly assigned to experimental groups. No samples or animals were excluded from the analysis, and no pre-established inclusion or exclusion criteria were applied. No blinding was performed.

### Measurement of guanine level

Cells were cultured in wells with 1640 supplemented with 10% dialyzed FBS. Guanine concentrations in cell lysates were then determined using GTPase-Glo™ Assay Kit (V7681 and V7682), following the manufacturer’s instructions. A Varioskan LUX Multimode Microplate Reader (Thermo Fisher Scientific) was used to measure the fluorescent intensity (Ex/Em = 535/587 nm).

### ^13^C-labeled metabolite analysis by LC–MS/MS

KYSE150 cells (5 × 10^6^) were washed twice with PBS and then cultured in glucose-free medium supplemented with 10% dialyzed fetal bovine serum (dialyzed FBS) and 25 mM uniformly 13C-labeled glucose (U-[^13^C]-glucose, Cambridge Isotope Laboratories, Cat# CLM1396) for 24 h. After incubation, cells were washed twice with cold PBS and resuspended in pre-chilled 80% methanol and 20% double-distilled water (ddH_2_O) for metabolite extraction. The abundance of purine-related metabolites incorporating ^13^C from glucose was subsequently analyzed by LC–MS/MS at LipidALL Technologies (Changzhou, China).

### Untargeted metabolomic analysis

Untargeted metabolomic analysis of 30 human esophageal cancer tissues and paired adjacent normal tissues was performed using liquid chromatography–tandem mass spectrometry (LC–MS/MS) (Vanquish, Orbitrap Exploris 120, Thermo Fisher Scientific) in collaboration with PANOMIX (Suzhou, China). The 30 paired tumor and adjacent normal tissues were obtained from The Affiliated Cancer Hospital of Zhengzhou University. Data processing was conducted using the ProteoWizard package (v3.0.8789), the R XCMS package (v3.12.0), the R package Ropls, and the MetaboAnalyst package. Metabolite identification was based on public databases, including HMDB, MassBank, LipidMaps, mzCloud, and KEGG spectral databases.

### Ubiquitination assay

Cells were transfected with HA-tagged ubiquitin, Flag-tagged USP5, and Myc-tagged IMPDH2 expression plasmids using Lipofectamine 2000 (Invitrogen) according to the manufacturer’s protocol. Forty-eight hours post-transfection, cells were treated with 10 μM MG132 for 6 h to inhibit proteasomal degradation.

Cells were then lysed in denaturing lysis buffer and boiled at 95 °C for 10 min to disrupt non-covalent interactions. The lysates were diluted tenfold with NP-40 lysis buffer and centrifuged at 12,000 rpm for 10 minutes at 4 °C to remove debris.

The supernatants were incubated with anti-Flag antibody overnight at 4 °C, followed by incubation with protein A/G agarose beads (Thermo Fisher) overnight at 4 °C. The beads were washed five times with NP-40 buffer and boiled in SDS loading buffer to elute the bound proteins. Samples were subjected to SDS-PAGE and analyzed by immunoblotting with an anti-HA antibody to detect the ubiquitinated forms of the target protein. Immunoprecipitation efficiency was verified by probing with anti-Flag or anti-target protein antibodies.

### Statistics and reproducibility

All experiments were independently performed at least three times. Sample sizes were determined based on previous studies and pilot experiments to ensure adequate statistical power. For animal studies, sample sizes were estimated based on prior experience and published literature to detect biologically meaningful differences. No formal statistical methods were used to predetermine sample size. Statistical analyses were performed using GraphPad Prism.

Normality of data distribution was assessed using the Shapiro-Wilk test, and homogeneity of variances was evaluated prior to parametric analyses. Data with normal distribution are presented as mean ± standard deviation (SD). Comparisons between two groups were conducted using paired or unpaired two-tailed Student’s t-tests, while comparisons among multiple groups were performed using one-way or two-way ANOVA followed by appropriate post hoc tests. For data that did not meet normality assumptions, non-parametric tests were applied, including the Mann-Whitney U test for two-group comparisons and the Kruskal-Wallis test for multiple-group comparisons. Overall survival was analyzed using Kaplan–Meier curves with the log-rank test. A *P* value < 0.05 was considered statistically significant. No samples or animals were excluded from the analysis.

## Results

### USP5 overexpression correlates with upregulated purine metabolism in ESCC

**Fig. 1:**
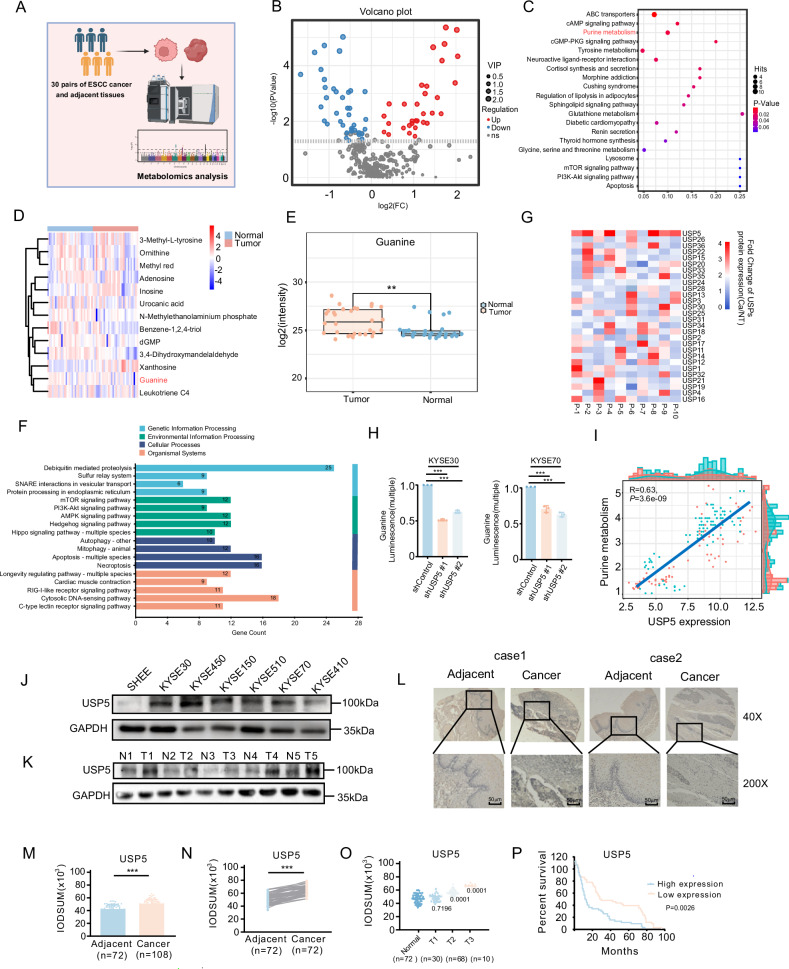
High USP5 Expression Is Associated with Purine Metabolism. **A** Schematic of the experimental design for untargeted metabolomic profiling of 30 pairs of ESCC tumor and adjacent non-tumor tissues. **B** Volcano plot of differential metabolites in human ESCC tumor and adjacent non-tumor tissues. **C** KEGG pathway enrichment analysis of different metabolites. **D** Heatmap of the metabolites in ESCC tumor and adjacent non-tumor tissues. **E** Guanine levels are significantly elevated in tumor tissues compared to adjacent normal tissues. **F** TCGA analysis of deubiquitination-mediated proteolysis activity in ESCC. **G** TCGA analysis of deubiquitinase expression levels in ESCC. **H** Quantification of guanine levels in ESCC cells after USP5 knockdown. **I** TCGA analysis of the association between USP5 expression and purine metabolism in ESCC. **J** Western blot analysis of USP5 protein levels in SHEE and ESCC cell lines. **K** Western blot analysis of USP5 protein levels in tumor tissues from 5 ESCC patients. **L** Representative immunohistochemical (IHC) staining images of USP5 in a commercial ESCC tissue microarray containing 66 pairs of tumor and adjacent normal tissue samples. **M**–**N** Quantification of USP5 protein levels by IHC in ESCC tissues and adjacent normal tissues, presented as mean IOD (integrated optical density). **O** IHC analysis of USP5 expression across different tumor stages (T1–T3) in ESCC tissues. **P** Kaplan–Meier survival curve analysis of the correlation between USP5 expression and overall survival in ESCC patients. Data are presented as mean ± SD. Statistical significance was assessed by paired two-tailed Student’s *t* test (**E**, **N**), unpaired two-tailed Student’s *t* test (**M**), log-rank test (**P**) or one-way ANOVA (**H**, **O**) as appropriate3. *p < 0.05; **p < 0.01; ***p < 0.001.

Tumors were characterized by aberrant metabolic reprogramming [27]. To investigate metabolic alterations in ESCC, we collected 30 paired tumor and adjacent normal tissue samples from ESCC patients undergoing surgical resection at The Affiliated Cancer Hospital of Zhengzhou University, and conducted untargeted metabolomics analysis (Fig. 1A). The results revealed widespread dysregulation of metabolites between tumor and adjacent normal tissues (Fig. 1B). The OPLS-DA score plot and volcano plots (Supplementary Fig. 1A, B) confirmed the robustness and reproducibility of the metabolomic analysis. KEGG pathway enrichment analysis indicated a marked enrichment of purine metabolism in ESCC tissues (Fig. 1C and Supplementary Fig. 1C, D). Further analysis revealed that the guanine, a purine metabolite, was significantly upregulated in tumor tissues compared to adjacent normal tissues (Fig. 1D, E), indicating enhanced guanine biosynthesis in ESCC. Previous studies have demonstrated that deubiquitinating enzymes (DUBs) play a critical role in tumor initiation and progression [28]. Given the marked dysregulation of purine metabolism observed in esophageal cancer, we hypothesized that this metabolic reprogramming might be regulated by DUBs.

Based on this, we analyzed the TCGA-ESCC cohort and found that biological processes associated with esophageal carcinogenesis were significantly enriched in ubiquitin-related pathways. Among these, ubiquitin-mediated proteolysis exhibited the highest enrichment score, suggesting that its dysregulation may play a pivotal role in the initiation and progression of esophageal cancer (Fig. 1F). Next, we analyzed the expression levels of USPs in esophageal cancer and adjacent normal tissues using the TCGA database, and found that USP5 exhibited the highest expression in tumor tissues (Fig. 1G). Next, we measured guanine levels following USP5 knockdown and found that guanine production was significantly suppressed upon USP5 depletion (Fig. 1H). When we mutated the catalytic site (C335) of USP5, its deubiquitinase activity was impaired, leading to reduced guanine production (Supplementary Fig. 1E). This indicates that USP5 mediates purine metabolism through its deubiquitinase function. To investigate the potential association between USP5 and purine metabolism, we analyzed their correlation using publicly available databases and found that USP5 expression was positively associated with purine metabolic (Fig. 1I). In addition, USP5 knockdown suppressed the proliferation of ESCC cell lines, whereas exogenous guanine supplementation rescued this inhibitory effect (Supplementary Fig. 1F). These results collectively indicate that deubiquitinases play a crucial role in regulating purine metabolism and tumor progression in esophageal cancer.

Next, we investigated the role of USP5 in the progression of ESCC. We first examined the expression of USP5 in ESCC cell lines and SV40-immortalized human esophageal epithelial cells (SHEE), and found that USP5 was highly expressed in ESCC cell lines (Fig. 1J). Western blot analysis of tumor tissues and matched adjacent normal tissues from five ESCC patients further confirmed a marked increase in USP5 protein levels in tumor samples (Fig. 1K). To further validate the high expression of USP5 in ESCC, we performed immunohistochemical (IHC) staining used a commercial tissue microarray containing 66 pairs of ESCC tumors and adjacent normal tissues (Fig. 1L). The results showed that USP5 expression was significantly elevated in ESCC tumor tissues compared to normal tissues and positively correlated with clinical grade (Fig. 1M–O). Moreover, high USP5 expression in ESCC patients was associated with poorer overall survival (Fig. 1P). These results demonstrate that USP5 is highly expressed in ESCC and closely associated with tumorigenesis and progression, potentially promoting tumor development by regulating purine metabolism, suggesting that USP5 may serve as a key driver of esophageal cancer and a promising therapeutic target.

### USP5 promotes ESCC proliferation and progression

**Fig. 2:**
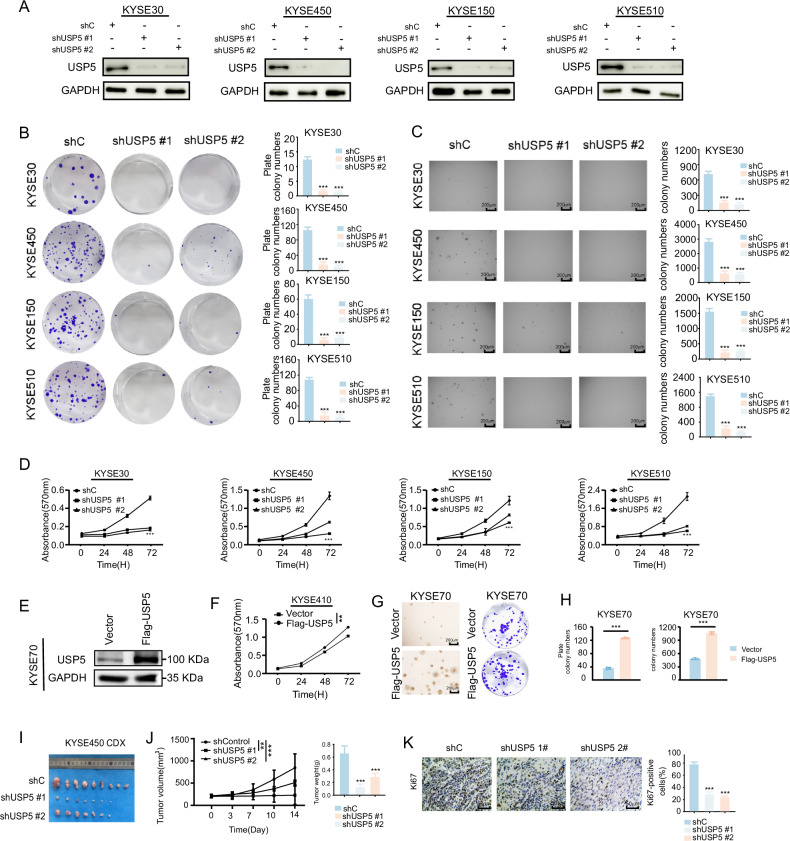
USP5 promotes ESCC cell proliferation and tumor growth in vitro and in vivo. **A** Western blot analysis of USP5 protein levels in KYSE30, KYSE450, KYSE150, and KYSE510 cells transfected with control shRNA (shC) or two independent USP5-targeting shRNAs (shUSP5#1, shUSP5#2). **B**, **C** Foci formation and soft agar assays evaluating the effects of USP5 knockdown on clonogenic potential of ESCC cells. **D** MTT assay assessing the impact of USP5 knockdown on ESCC cell proliferation. **E**—**H** Effects of USP5 overexpression on in vitro proliferation and colony formation capacity of ESCC cells. **I** In vivo CDX mouse models established using and KYSE450 cells. **J** Measurement of tumor volume and weight following USP5 knockdown. **K** Immunohistochemical staining showing the expression levels of Ki67. Data are presented as mean ± SD. Statistical significance was assessed by unpaired two-tailed Student’s *t* test (**H**) two-way ANOVA (**D**, **F**) or one-way ANOVA (**B**, **C**, **J**, **K**) as appropriate. *p < 0.05; **p < 0.01; ***p < 0.001.

To investigate the role of USP5 in ESCC, we knocked down USP5 (Fig. 2A), which significantly reduced cell proliferation in the foci formation and soft agar assays (Fig. 2B, C). Through MTT measurement, after knocked down USP5, the cell proliferation of these cell lines was significantly reduced (Fig. 2D). On the contrary, overexpression of USP5 increased cell proliferation and clonality (Fig. 2E–H). In order to further investigate the effect of USP5 on ESCC progression in vivo, we used a CDX model and subcutaneously inoculated KYSE 450 cells with USP5 knockdown into nude mice. The results indicated that knocked down USP5 significantly reduced tumor growth in the CDX model (Fig. 2I). The volume and weight of tumors in the shUSP5 group were significantly reduced (Fig. 2J), and IHC results showed that knockdown of USP5 markedly decreased Ki67 levels (Fig. 2K). In conclusion, this study demonstrated that USP5 is highly expressed in ESCC and promotes cell proliferation, colony formation, and tumor progression.

### USP5 drives purine biosynthesis to support ESCC growth

**Fig. 3:**
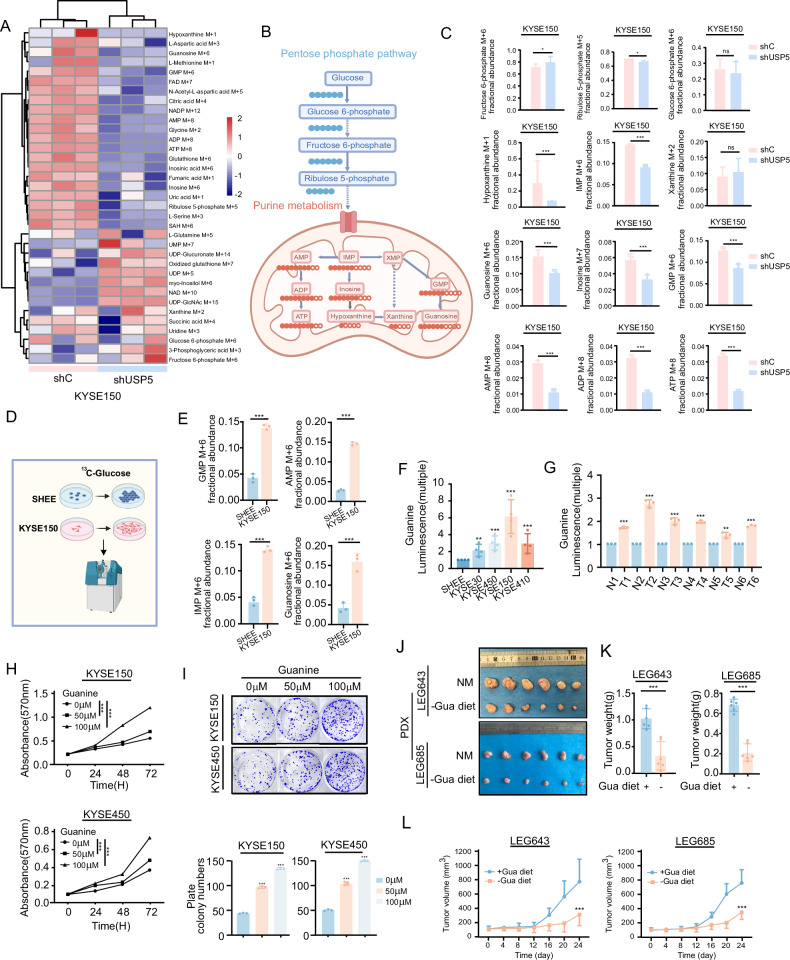
USP5 drives purine biosynthesis to support ESCC growth. **A** Heatmap enrichment analysis of metabolic flux data following USP5 knockdown. **B** Schematic of ^13^C -Glucose labeled metabolic flux analysis in KYSE150 cells after USP5 knockdown. **C** ^13^C metabolic flux analysis of metabolite levels in the purine metabolism pathway following USP5 knockdown. **D** Schematic diagram of ^13^C-Glucose tracing experiment in SHEE and KYSE150 cells. **E** Analysis of ^13^C-labeled purine intermediates in SHEE and KYSE150 cells. **F** Measurement of guanine levels in various ESCC cell lines and SHEE cells. **G** Measurement of guanine levels in patient-derived tumor and adjacent normal tissues. **H**, **I** MTT assay and colony formation assay of ESCC cells treated with exogenous purine. **J** Tumors from ESCC PDX models under purine-restricted and control diets. NM, normal diet; Gua diet +/− , with or without guanine supplementation. **K**, **L** Tumor weight and tumor volume in control and purine-restricted diet groups. Data are presented as mean ± SD. Statistical significance was assessed by unpaired two-tailed Student’s *t* test (**C**, **E**, **G**, **K**) two-way ANOVA (**H**, **L**) or one-way ANOVA (**F**, **I**) as appropriate. *p < 0.05; **p < 0.01; ***p < 0.001.

To validate the association between USP5 and purine metabolism identified from database analysis, we performed ¹³C-based metabolic flux tracing experiments. Metabolic flux analysis demonstrated that knocking down USP5 in KYSE150 cells significantly perturbed multiple metabolic pathways, among which purine metabolism was most prominently affected (Fig. 3A). Based on the schematic analysis of the de novo purine biosynthesis pathway (Fig. 3B), USP5 knockdown led to the suppression of multiple metabolites within the purine metabolic cascade—including hypoxanthine, Inosine Monophosphate (IMP), guanosine, inosine, Guanosine Monophosphate (GMP), Adenosine Monophosphate (AMP), Adenosine diphosphate (ADP) and Adenosine triphosphate (ATP) (Fig. 3C). Metabolic flux analysis indicates that USP5 plays a critical role in ESCC by regulating purine metabolism, highlighting its importance in tumor metabolic reprogramming.

To validate the significant upregulation of guanine in ESCC tissues, We utilized SHEE and KYSE150 esophageal cancer cells with ^13^C-labeled glucose to trace the intracellular metabolic fate of carbon atoms (Fig. 3D). Results showed that compared to the SHEE, the ESCC cell line KYSE150 exhibited increased incorporation of labeled carbon into purine metabolites such as GMP, AMP, IMP and Guanosine (Fig. 3E), indicated elevated de novo purine biosynthesis in cancer cells. We further quantified intracellular Guanine levels across various ESCC cells and SHEE. Consistent with the metabolic flux data, Guanine levels were markedly elevated in ESCC cell lines relative to SHEE cells (Fig. 3F). To verify the reliability of this analysis, we measured guanine levels in esophageal tumor and adjacent tumor tissues, and found that guanine content was significantly elevated in tumor tissues compared to adjacent tissues. (Fig. 3G). This finding indicated that guanine was persistently upregulated in ESCC, suggesting its potential role as a metabolic driver in tumor progression and highlighting its value as a prospective therapeutic target.

To further investigate the impact of guanine metabolism on ESCC progression, KYSE150 and KYSE450 cells were supplemented with guanine. Both MTT and colony formation assays demonstrated that guanine supplementation significantly enhanced ESCC cell proliferation (Fig. 3H, I), further underscoring the critical role of guanine metabolism, in supporting tumor growth. Similarly, we established a patient-derived xenograft (PDX) mouse model and implemented a free-purine diet intervention. The results showed that compared with the control group fed a normal diet (NM), free-purine diet significantly suppressed tumor growth (Fig. 3J), as evidenced by notable reductions in both tumor volume and weight (Fig. 3K, L), indicated that free-purine diet in vivo can effectively inhibit tumor development. Collectively, these findings demonstrated that guanine metabolism was profoundly reprogrammed in ESCC and plays a critical role in supporting tumor progression.

### USP5 stabilizes IMPDH2 via its deubiquitinase activity

**Fig. 4:**
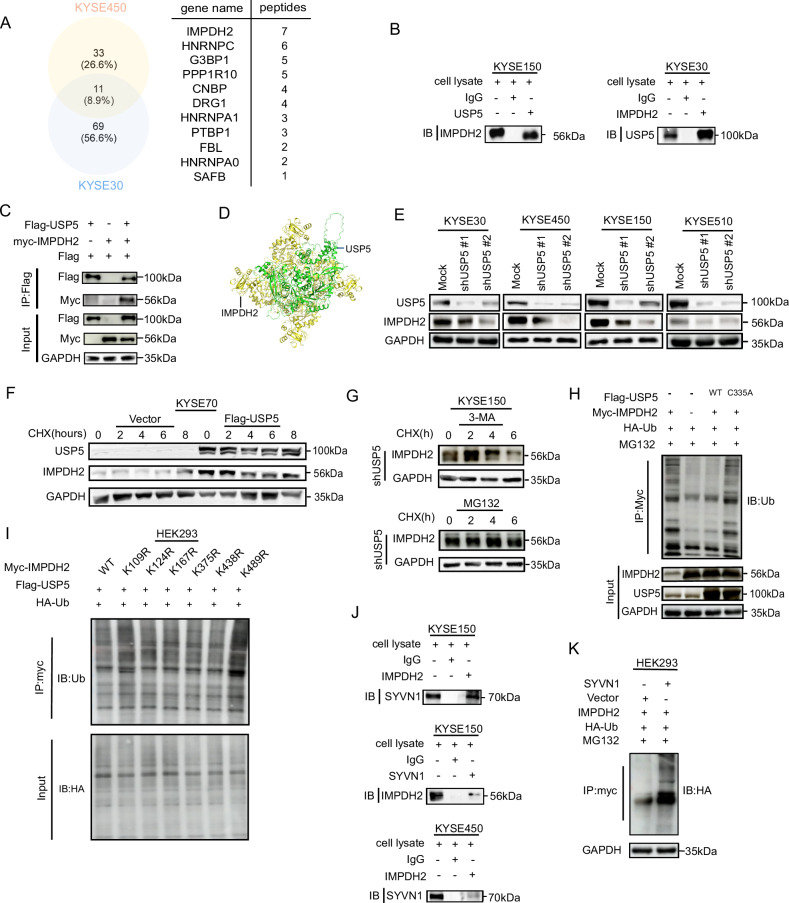
IMPDH2 is a downstream target of the deubiquitinase USP5. **A** MS analysis identified proteins commonly enriched in KYSE450 and KYSE30 cells. **B**, **C** Endogenous and exogenous co-IP confirmed the interaction between USP5 and IMPDH2. **D** Molecular docking of USP5 with IMPDH2. **E** IMPDH2 protein levels were assessed by WB after USP5 knockdown. **F** IMPDH2 protein stability was assessed by WB after USP5 overexpression, and CHX treatment at various time points. **G** KYSE150 cells were treated with 3-MA or MG132 after USP5 knockdown, and IMPDH2 stability was analyzed by WB after CHX treatment. **H** Flag-USP5 WT or Flag-USP5 C335A plasmids were transfected into HEK293 cells, and IMPDH2 ubiquitination was assessed by Western blot after 6 h of MG132 treatment. **I** In HEK293 cells, Myc-IMPDH2, Myc-IMPDH2-K109R, Myc-IMPDH2-K124R, Myc-IMPDH2-K167R, Myc-IMPDH2-K375R, Myc-IMPDH2-K438R, Myc-IMPDH2-K489R plasmids were transfected, followed by MG132 treatment, and IMPDH2 ubiquitination levels were assessed by Western blot. **J** Endogenous and exogenous co-IP with SYVN1 and IMPDH2 antibodies was performed by WB in KYSE150 and KYSE450 cells. **K** SYVN1 plasmid was transfected into HEK293 cells, followed by MG132 treatment, and IMPDH2 ubiquitination levels were assessed by Western blot.

To elucidate the molecular mechanism by which USP5 regulates guanine, we conducted LC-MS/MS analysis of USP5 immunoprecipitated from KYSE450 and KYSE30 cells. The analysis identified 44 and 80 proteins enriched in the respective cell lysates, with 11 proteins common to both cell lines (Fig. 4A). Notably, IMPDH2 exhibited the highest number of detected peptides and serves as a key rate-limiting enzyme in de novo guanine synthesis.

To systematically evaluate these candidates, we experimentally validated all identified proteins for their potential interaction with USP5. Among them, only IMPDH2 demonstrated a specific and reproducible association with USP5 (Supplementary Fig. 2A). To validate the interaction between USP5 and IMPDH2, we performed both endogenous and exogenous co-immunoprecipitation assays. These results consistently demonstrated a direct interaction between USP5 and IMPDH2 in cells (Fig. 4B, C and Supplementary Fig. 2B, C). We also performed immunofluorescence staining followed by confocal microscopy analysis, the results demonstrated a clear co-localization of IMPDH2 and USP5 in ESCC cell lines (Supplementary Fig. 2D). Furthermore, molecular docking analysis was conducted to predict the potential binding domains between IMPDH2 and USP5 (Fig. 4D), provided structural insights into their interaction and identifying possible regions that mediate their association. Subsequently, to explore the regulatory relationship between USP5 and IMPDH2, we knocked down USP5 used short hairpin RNA (shRNA) in ESCC cell lines. Quantitative PCR (qPCR) analysis showed that USP5 knockdown did not significantly affect IMPDH2 mRNA levels, while the protein levels of IMPDH2 were markedly decreased, indicating that USP5 regulates IMPDH2 expression primarily at the post-translational level (Supplementary Fig. 2E and Fig. 4E).

USP5 is a widely reported deubiquitinating enzyme that regulates the stability and function of substrate proteins by removing ubiquitin modifications. Given this role, we investigated whether USP5 regulates the stability of IMPDH2 and thereby contributes to the progression of ESCC. To assess the impact of USP5 on IMPDH2 protein stability, we employed cycloheximide (CHX), an inhibitor of protein synthesis, to assess IMPDH2 degradation kinetics. Overexpression of USP5 markedly enhanced the stability of IMPDH2 following CHX treatment in a time-dependent manner (Fig. 4F). In contrast, USP5 knockdown accelerated IMPDH2 degradation, indicating that USP5 positively regulates IMPDH2 protein stability (Supplementary Fig. 2F). To further delineate the degradation pathway involved, we employed MG132, a proteasome inhibitor, and 3-methyladenine (3-MA), an autophagy inhibitor. Notably, MG132, but not 3-MA, reversed CHX-induced degradation of IMPDH2, supporting the conclusion that USP5 regulates IMPDH2 stability predominantly through the ubiquitin–proteasome pathway (Fig. 4G). Cys335 serves as the catalytic residue of USP5 in its deubiquitinating activity [29]. The C335A mutant plasmid of USP5, which lacks deubiquitinase activity, abrogated the WT USP5-mediated deubiquitination and protein upregulation of IMPDH2, supported the notion that USP5 stabilizes IMPDH2 through its enzymatic activity (Fig. 4H). While overexpression of USP5 led to a decrease in the ubiquitination level of IMPDH2, indicated that USP5 enhanced IMPDH2 stability by reducing its ubiquitination (Supplementary Fig. 2G). Consistently, knocked down of USP5 resulted in increased ubiquitination of IMPDH2 and reduced stability of the IMPDH2 protein (Supplementary Fig. 2H).

To identify the specific type of ubiquitin linkage removed by USP5, we introduced various ubiquitin chain mutants (K6, K11, K27, K29, K33, K48, K63) into lenti-293 cells. The results demonstrated that USP5 selectively recognizes and cleaves K48-linked polyubiquitin chains on IMPDH2 (Supplementary Fig. 2I). Similarly, the same results were obtained when we performed reverse validation with ubiquitin mutant plasmids (Supplementary Fig. 2J). To investigate the specific ubiquitination sites on IMPDH2 targeted by USP5 for deubiquitination, we predicted the potential ubiquitination sites of IMPDH2 using the ubiquitination site prediction website UbiBrowser-Database Commons (cncb.ac.cn) and performed site-directed mutagenesis. Experimental validation confirmed that lysine 489 (K489) was the critical site at which USP5 removes K48-linked ubiquitin chains (Fig. 4I). To investigate the E3 ligase that facilitates USP5-mediated deubiquitination of IMPDH2, we subsequently utilized the UbiNet 2.0 platform (cuhk.edu.cn) to predict potential E3 ligases interacting with IMPDH2. Our experimental validation confirmed that only the E3 ligase SYVN1 physically associates with IMPDH2 (Fig. 4J) and mediates its ubiquitination-dependent proteasomal degradation (Fig. 4K). In conclusion, USP5 mediates the deubiquitination of IMPDH2 by specifically targeting the K489 site. This process involves the removal of K48-linked polyubiquitin chains from the lysine residue at position 489. Consequently, USP5 prevents the proteasomal degradation of IMPDH2, thereby stabilizing its protein expression.

To verify whether IMPDH2 was aberrantly expressed in ESCC, we analyzed data from TCGA. The analysis revealed that IMPDH2 was broadly overexpressed across multiple cancer types, including ESCC, and its expression correlates with advanced clinical stage (Supplementary Fig. 3A, B). Consistently, IMPDH2 protein levels were highly expressed in ESCC cell lines and primary tumor tissues. (Supplementary Fig. 3C). Immunohistochemistry (IHC) analysis performed on a commercially available ESCC tissue microarray (*n* = 66 paired samples) further validated the elevated expression of IMPDH2 protein in tumor tissues. Higher expression levels were associated with increased lymph node metastasis, larger tumor size, and more advanced stage (Supplementary Fig. 3D–G). Moreover, high expression of IMPDH2 was correlated with poorer overall survival in ESCC patients (Supplementary Fig. 3H). The oncogenic role of IMPDH2 was further validated by its overexpression and knockdown. Specifically, in KYSE410 cells with relatively low endogenous IMPDH2 levels, enforced overexpression markedly promoted cell proliferation and colony-forming ability(Supplementary Fig. 4A–D). Conversely, knockdown of IMPDH2 via shRNA in KYSE30, KYSE450, and KYSE510 cell lines significantly impaired cell proliferation, as measured by MTT assay (Supplementary Fig. 4E), and reduced anchorage-dependent and anchorage-independent growth, as shown in foci formation and soft agar colony assays (Supplementary Fig. 4F, G). In CDX models established with KYSE150 and KYSE450 cells, IMPDH2 knockdown dramatically suppressed tumor growth in vivo, with reduced tumor volume and weight (Supplementary Fig. 4H). These findings indicated that IMPDH2 was highly expressed in ESCC, and its knockdown suppressed ESCC proliferation.

### USP5–IMPDH2 axis regulated purine metabolism and contributed to ESCC progression

**Fig. 5:**
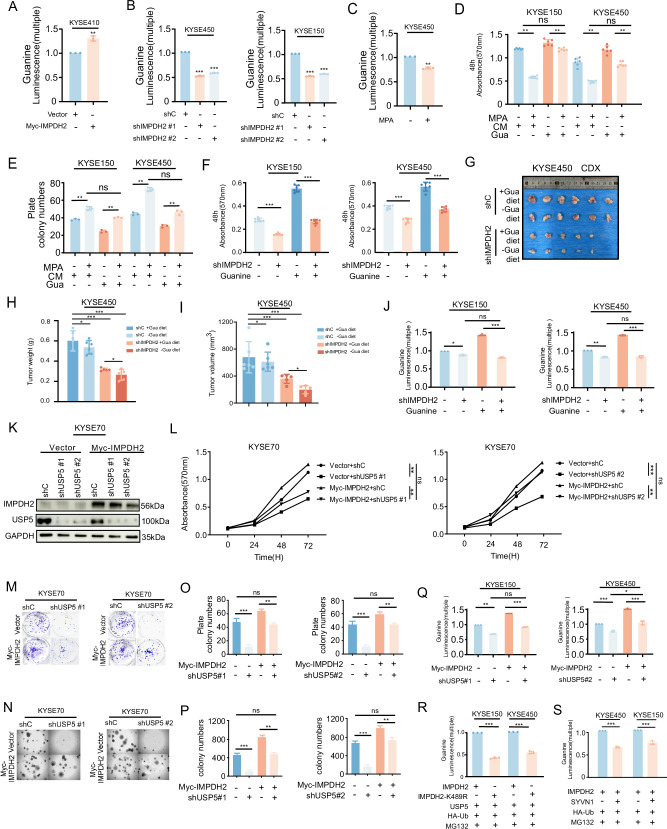
USP5–IMPDH2 axis regulated purine metabolism and contributed to ESCC progression. **A** Measurement of guanine levels in KYSE410 cells following IMPDH2 overexpression. **B** Measurement of guanine levels in KYSE450 and KYSE150 cells after IMPDH2 knockdown. **C** Guanine levels in KYSE450 cells after treatment with MPA (mycophenolic acid). **D**, **E** MTT and foci formation assays in KYSE150 and KYSE450 cells treated with MPA and supplemented with exogenous guanine. **F** MTT assay was conducted to measure the proliferation in KYSE150 and KYSE450 cells after IMPDH2 knockdown with exogenous guanine supplementation. **G**–**I** CDX model combining IMPDH2 knockdown and free-purine diet; tumor weight (**H**) and volume (**I**) measurements. **J** In KYSE150 and KYSE450 cells with IMPDH2 knockdown, exogenous guanine was added, and guanine levels were measured. **K** Western blot validation of USP5 knockdown and IMPDH2 overexpression in KYSE70 cells. **L** MTT assay evaluating the effect of USP5 knockdown and IMPDH2 overexpression on cell proliferation in KYSE70 cells. **M**–**P**. Foci formation and soft agar assays assessing the impact of USP5 knockdown and IMPDH2 overexpression on clonogenic capacity of ESCC cells. **Q** In USP5-knockdown KYSE150 and KYSE450 cells, Myc-IMPDH2 plasmid was transfected, and guanine levels were measured. **R** In USP5-knockdown KYSE150 and KYSE450 cells, Flag-USP5, Myc-IMPDH2, and Myc-IMPDH2-K489R plasmids were transfected, followed by MG132 treatment, and guanine levels were measured. **S** In KYSE150 and KYSE450 cells, Flag-SYVN1 and Myc-IMPDH2 plasmids were transfected, followed by MG132 treatment, and guanine levels were measured. Data are presented as mean ± SD. Statistical significance was assessed by unpaired two-tailed Student’s *t* test (**A**, **C**, **R**, **S**) two-way ANOVA (**L**) or one-way ANOVA (**B**, **D**, **E**, **F**, **H**, **I**, **J**, **O**, **P**, **Q**) as appropriate. *p < 0.05; **p < 0.01; ***p < 0.001.

To further determine whether IMPDH2 was responsible for the elevated guanine levels observed in ESCC. We overexpressed of IMPDH2 and found that IMPDH2 markedly increased guanine concentration, further confirmed its pivotal role in de novo guanine synthesis in ESCC cells (Fig. 5A). Conversely, knockdown of IMPDH2 or treatment with its inhibitor mycophenolic acid (MPA) significantly reduced intracellular guanine levels (Fig. 5B, C).These results suggested that IMPDH2 was a key driver of guanine accumulation in ESCC.

We next sought to determine whether IMPDH2-mediated guanine accumulation functionally contributes to the proliferative capacity of ESCC cells, we co-treated cells with guanine and MPA, and compared to control medium (CM), exogenous guanine supplementation effectively rescued the inhibitory effects of MPA on ESCC cell proliferation and colony formation (Fig. 5D, E). Similarly, exogenous guanine reversed the proliferation defects caused by IMPDH2 knockdown (Fig. 5F). In vivo, combining dietary guanine restriction with IMPDH2 knockdown in a cell-derived xenograft (CDX) model resulted in the greatest suppression of tumor growth, as evidenced by reduced tumor volume and weight (Fig. 5G–I). Exogenous guanine supplementation partially restored guanine levels in IMPDH2-knockdown cells (Fig. 5J), indicating that IMPDH2-driven proliferation depends on guanine metabolism. To investigate the effect of the USP5-IMPDH2 axis on ESCC cell proliferation, we performed rescue experiments by knocking down USP5 and overexpressing IMPDH2 in ESCC cells, followed by in vitro proliferation assays (Fig. 5K). Knockdown of USP5 significantly suppressed cell proliferation, whereas overexpression of IMPDH2 can significantly salvage this inhibitory effect (Fig. 5L). Similarly, Foci Formation and Soft Agar corroborated the conclusion (Fig. 5M–P). These results demonstrated that USP5 promoted ESCC cell proliferation largely through the stabilization of IMPDH2, highlighted the USP5–IMPDH2 axis as a potential metabolic vulnerability for therapeutic intervention. Furthermore, we assessed the metabolic consequences of USP5-mediated regulation of IMPDH2, focusing on guanine biosynthesis. Similarly, USP5 knockdown impaired guanine synthesis, while IMPDH2 overexpression was able to restore guanine levels (Fig. 5Q). Mutation of the K489 site on IMPDH2, the specific deubiquitination target of USP5, abrogated the stabilizing effect of USP5 and led to decreased guanine levels (Fig. 5R). After co-expressed E3 ligase SYVN1, which mediates IMPDH2 ubiquitination and degradation, also significantly reduced guanine production (Fig. 5S). These findings demonstrate that IMPDH2 drives ESCC progression by regulating de novo guanine synthesis.

Considering that transporters may also influence intracellular purine levels, we investigated the role of purine transporters SLC28A2, SLC28A3, SLC29A1, and SLC29A2 in this context (Supplementary Fig. 5A). We first examined the expression levels of these transporters in ESCC using data from The Cancer Genome Atlas (TCGA) and found that SLC28A3, SLC29A1, and SLC29A2 were significantly upregulated in cancer tissues compared to adjacent normal tissues (Supplementary Fig. 5B). To further investigate their roles in guanine transport, we overexpressed these three transporters in ESCC cell lines and measured intracellular guanine content. Only SLC29A1 increased intracellular guanine levels, indicated SLC29A1 was the primary guanine transporter in ESCC cells. (Supplementary Fig. 5C). To investigate the functional role of SLC29A1, we assessed SLC29A1 expression in SHEE and ESCC lines. We found that SLC29A1 was highly expressed in all ESCC cell lines (Supplementary Fig. 5D). Analysis of TCGA data revealed that SLC29A1 expression correlates with the grade and stage of ESCC, suggesting its potential role in tumor progression (Supplementary Fig. 5E). Through immunohistochemistry (IHC) using a commercially available ESCC tissue microarray (*n* = 72 pairs), we further confirmed that SLC29A1 protein levels were elevated in tumor tissues (Supplementary Fig. 5F, G). Higher expression levels were associated with increased lymph node metastasis and more advanced stage (Supplementary Fig. 5H). Moreover, high expression of SLC29A1 was correlated with poorer overall survival in ESCC patients (Supplementary Fig. 5I). Next, we knocked down SLC29A1 in ESCC cells and observed a significant inhibition of cell proliferation, further supported the functional role of SLC29A1 in cancer cell growth (Supplementary Fig. 5J). Moreover, knockdown of SLC29A1 abolished the proliferative effect induced by guanine supplementation, as no significant difference in cell proliferation was observed between cells lacking SLC29A1 with or without guanine treatment (Supplementary Fig. 5K). To confirm the role of SLC29A1 in guanine transport, we supplemented cells with additional guanine after SLC29A1 knockdown and measured intracellular and extracellular guanine levels (Supplementary Fig. 5L, M). The results demonstrated that guanine supplementation failed to elevate intracellular guanine levels in SLC29A1 knockdown cells, indicated that SLC29A1 was required for guanine uptake in ESCC cells. These results indicate that guanine transport mediated by SLC29A1 is essential for proliferation induced by guanine in ESCC cells. We further analyzed the correlations among USP5, IMPDH2, and SLC29A1(Supplementary Fig. 5N–P). The results showed a significant positive correlation between USP5 and IMPDH2, whereas SLC29A1 showed no correlation with either protein. These findings indicate that purine de novo synthesis and purine transport may represent two independent regulatory pathways. Together, these findings established that SLC29A1 was a key transporter responsible for guanine uptake in ESCC cells and plays a significant role in supporting tumor cell growth.

Furthermore, our results systematically reveal the critical role of the USP5–IMPDH2 axis in controlling purine biosynthesis, highlighting its importance in metabolic reprogramming and ESCC progression.

### Mebendazole, a USP5 inhibitor, inhibited ESCC tumor growth in vitro and in vivo

**Fig. 6:**
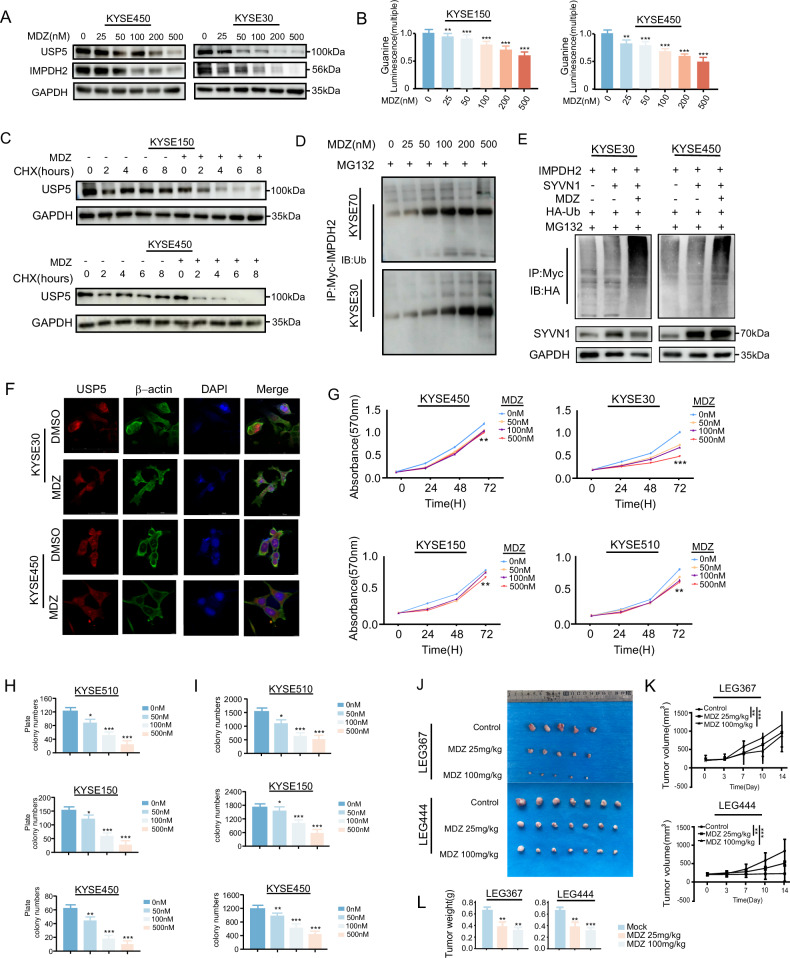
Mebendazole acts as a targeted inhibitor of USP5 to inhibit the growth of ESCC tumors. **A** Western blot analysis of USP5 and IMPDH2 protein expression after MDZ treatment. MDZ represents Mebendazole. **B** Guanine levels were measured in KYSE150 and KYSE450 cells after 48 hours of MDZ treatment. **C** USP5 protein stability was assessed in KYSE150 and KYSE450 cells after MDZ treatment followed by CHX treatment. **D** In KYSE70 and KYSE30 cells, MDZ treatment followed by MG132 treatment was performed, and IMPDH2 ubiquitination levels were assessed by Western blot. **E** In KYSE450 and KYSE30 cells, SYVN1 was transfected, followed by MDZ (Mebendazole) treatment and MG132 treatment, and IMPDH2 ubiquitination levels were assessed by Western blot. **F** Immunofluorescence staining was performed to detect USP5 protein levels after Mebendazole treatment. **G** MTT assay was performed to assess cell proliferation levels of KYSE30, KYSE450, KYSE150, and KYSE510 cells after MDZ treatment. **H**, **I** Foci formation and soft agar assays were performed to assess the colony formation ability of KYSE30, KYSE450, KYSE150, and KYSE510 cells after MDZ treatment. **J** Tumor images from the PDX model after intragastric administration of MDZ. **K**, **L** Tumor weight and tumor volume after MDZ (Mebendazole) treatment. Data are presented as mean ± SD. Statistical significance was assessed by two-way ANOVA (**G**, **K**) or one-way ANOVA (**B**, **H**, **I**, **L**) as appropriate. *p < 0.05; **p < 0.01; ***p < 0.001.

Mebendazole, a commonly used antipest drug, has recently been reported as a USP5 inhibitor to inhibit tumor growth [30]. To assess the therapeutic potential of targeting USP5 and to explore metabolism-based treatment strategies for ESCC, we investigated the effects of Mebendazole on the USP5–IMPDH2 axis. Western blot analysis revealed a dose-dependent decrease in USP5 protein levels, accompanied by a similar reduction in IMPDH2 expression (Fig. 6A). Notably, quantitative PCR analysis showed that Mebendazole treatment did not affect the mRNA levels of USP5 or IMPDH2, indicated that the downregulation occurs at the post-transcriptional level (Supplementary Fig. 6A). To evaluate the effect of Mebendazole on guanine biosynthesis, KYSE150 and KYSE450 cells were treated with Mebendazole. A dose-dependent reduction in guanine levels was observed in both cell lines (Fig. 6B). Moreover, co-treatment with CHX and Mebendazole demonstrated that USP5 protein turnover was accelerated, consistent with enhanced degradation of USP5 (Fig. 6C). Following proteasome inhibition with MG132, we observed a concentration-dependent increase in IMPDH2 ubiquitination upon Mebendazole treatment, suggesting that the drug facilitates USP5-mediated IMPDH2 degradation (Fig. 6D). After co-expressed the E3 ligase SYVN1, the addition of Mebendazole would significantly increase IMPDH2 ubiquitization(Fig. 6E). In addition, immunofluorescence staining revealed a significant reduction in USP5 protein levels upon Mebendazole administration, as evidenced by diminished green fluorescence intensity (Fig. 6F). MTT assay showed that cell proliferation was inhibited after addition of Mebendazole (Fig. 6G). And after added Mebendazole, anchorage-dependent growth decreased as shown in the decrease in foci formation and soft agar experiment(Fig. 6H, I and Supplementary Fig. 6B, C). Similarly, PDX experiments in vivo have shown that tumor growth was inhibited after intragastric administration of Mebendazole in mice (Fig. 6J–L).

In summary, Mebendazole targeted USP5 and modulated the USP5-IMPDH2 axis, thereby impairing guanine biosynthesis. This inhibition disrupted a key metabolic pathway essential for tumor cell survival, ultimately led to the suppression of ESCC initiation and progression.

### Combined treatment with Mebendazole and Oxaliplatin enhanced therapeutic efficacy in ESCC

**Fig. 7:**
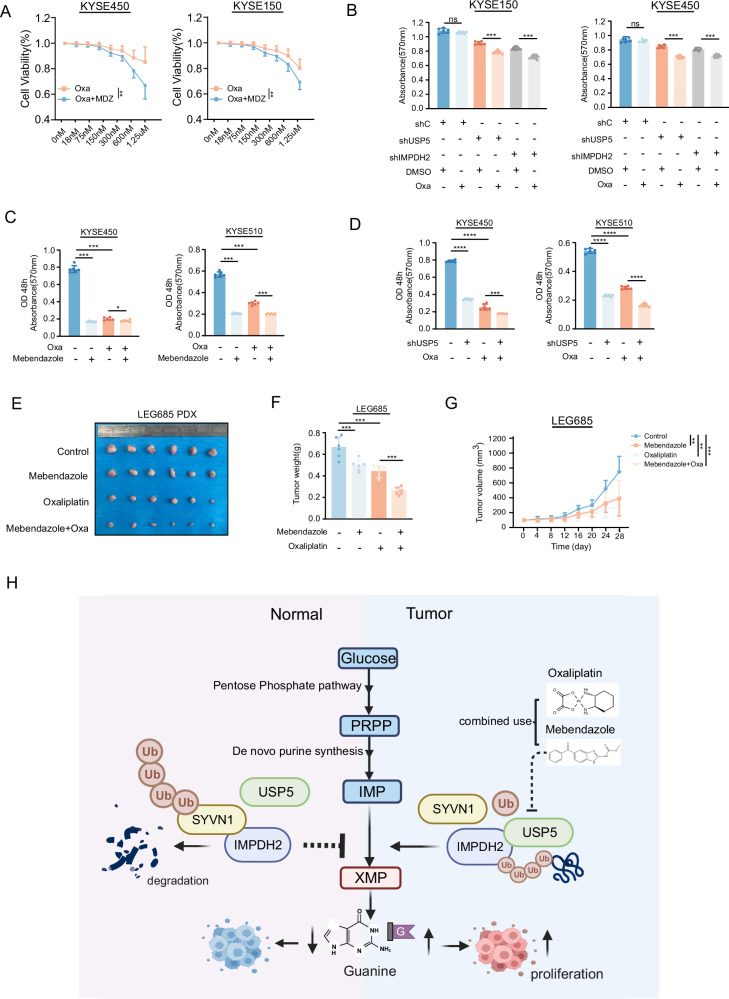
Inhibition of the USP5/IMPDH2 axis enhances the sensitivity of ESCC cells to Oxaliplatin. **A** MTT assay was performed to assess the proliferation of KYSE150 and KYSE450 cells after combined treatment with MDZ and Oxa. Oxa represents Oxaliplatin. **B** The sensitivity to Oxa was assessed in KYSE150 and KYSE450 cells after USP5 or IMPDH2 knockdown. **C**. MTT assay was assessed in KYSE450 and KYSE510 cells treated with a combination of MDZ and Oxa. **D** MTT assay was assessed in KYSE450 and KYSE510 cells treated with a combination of USP5 knockdown and Oxa. **E**–**G** The PDX model was used to evaluate the combined treatment of MDZ (25 mg/kg) and Oxa (5 mg/kg) (**E**), with tumor weight (**F**) and tumor volume (**G**) measured and analyzed. **H** Schematic diagram of the mechanism by which USP5 stabilizes IMPDH2 expression and promotes guanine biosynthesis. The USP5-targeting agent mebendazole enhances oxaliplatin sensitivity when used in combination therapy. Data are presented as mean ± SD. Statistical significance was assessed by two-way ANOVA (**A**, **G**) or one-way ANOVA (**B**, **C**, **D**, **F**) as appropriate. *p < 0.05; **p < 0.01; ***p < 0.001.

Oxaliplatin is a key chemotherapeutic agent commonly used in neoadjuvant therapy for esophageal cancer, Mebendazole has been shown to effectively inhibit the initiation and progression of the disease by targeting USP5-IMPDH2. To evaluate whether Mebendazole can act as a sensitizer to Oxaliplatin in neoadjuvant therapy for ESCC, we measured cell viability and found that co-treatment with Mebendazole markedly increased the sensitivity of ESCC cells to Oxaliplatin (Fig. 7A). Similarly, knockdown of either USP5 or IMPDH2 increased sensitivity to Oxaliplatin (Fig. 7B). Furthermore, combined treatment with Mebendazole or USP5 knockdown followed by Oxaliplatin treatment in KYSE450 and KYSE510 cells inhibited ESCC proliferation more effectively than Oxaliplatin alone (Fig. 7C, D). Moreover, in patient-derived xenograft (PDX) models, co-administration of Mebendazole significantly potentiated the antitumor effect of Oxaliplatin, further validated it can increase sensitivity of Oxaliplatin in vivo (Fig. 7E–G). In summary, the combination of Mebendazole with Oxaliplatin enhances the therapeutic efficacy in ESCC. This synergistic effect potentiates the anticancer activity of Oxaliplatin, improving its effectiveness in chemotherapy.

## Discussion

Ubiquitination is a fundamental post-translational modification that regulates protein stability [31], activity [32], and cellular signaling [33], playing critical roles in tumor initiation and progression. Dysregulation of ubiquitin ligases and deubiquitinases has been widely implicated in multiple cancer types, where they often modulate oncogenic pathways [34] and contribute to malignant phenotypes [35]. Despite these advances, the functional significance of ubiquitination in ESCC remains largely unexplored [36]. Our study provides novel evidence that USP5 can significantly impact tumor biology in ESCC, suggesting that USP5 as a critical regulator of guanine metabolism in ESCC. Mechanistically, USP5 deubiquitinates and stabilizes IMPDH2, the rate-limiting enzyme in guanine nucleotide biosynthesis, thereby enhancing purine synthesis and promoting tumor growth. These findings not only uncover a previously unrecognized USP5–IMPDH2 axis that drives metabolic reprogramming in ESCC, but also highlight USP5 as a promising therapeutic target for disrupting guanine metabolism in this malignancy.

IMPDH2 is a key metabolic enzyme that links ubiquitin signaling to purine biosynthesis in ESCC [37]. As the rate-limiting enzyme in guanine nucleotide synthesis, IMPDH2 not only fulfills the high proliferative demands of cancer cells but also exemplifies metabolic reprogramming as a critical hallmark of esophageal cancer development [26, 38]. Nevertheless, this study has several limitations. The in vivo validation of IMPDH2-dependent metabolic flux was confined to xenograft models and has not fully addressed the metabolic heterogeneity of patient tumors. Future studies should incorporate multi-omics analyses, patient-derived organoids, and clinical cohort validation to facilitate the translation of these mechanistic insights into practical therapeutic strategies.

The abnormal accumulation of guanine observed in ESCC may be attributed not only to enhanced de novo synthesis but also to dysregulation of nucleoside transport. Previous studies have identified several purine transporters, including SLC28A2, SLC28A3, SLC29A1, and SLC29A2 [39, 40], which mediate the cellular uptake and exchange of purine nucleosides and thereby contribute to the maintenance of intracellular nucleotide pools [41]. Alterations in the expression or activity of these transporters could influence guanine availability and metabolic homeostasis, potentially promoting purine accumulation and supporting the metabolic demands of rapidly proliferating tumor cells [42, 43]. Thus, dysregulated purine transport may represent an additional mechanism underlying metabolic reprogramming in ESCC.

Mebendazole, a traditional anthelmintic drug, has recently attracted attention for its antitumor activity demonstrated in various cancer models [44]. Its well-established safety profile and long history of clinical use provide a solid foundation for drug repurposing [45]. In this study, we found that Mebendazole may exert its anticancer effect by targeting USP5, thereby regulating the stability of IMPDH2 and disrupting purine metabolism, ultimately suppressing the proliferation of ESCC cells. Moreover, Mebendazole enhances the sensitivity of tumor cells to chemotherapeutic agents such as Oxaliplatin, showing promising potential for combinational therapy. Several clinical trials are currently underway to evaluate its antitumor efficacy in different cancer types [46], and it holds significant promise for clinical translation as a metabolic-targeting therapeutic agent in ESCC and other solid tumors. It is noteworthy that mebendazole has been reported to exert antitumor effects through multiple mechanisms. For example, MBZ can destabilize microtubules and induce mitotic arrest and apoptosis in cancer cells, consistent with its classic mode of action as a benzimidazole compound [47]. In addition, MBZ has been shown to inhibit Hedgehog signaling [48], suppress the USP5/c-Maf axis in myeloma [49], interfere with DNA unwinding complexes [50], and disrupt metabolic processes including glucose metabolism gene expression [51]. Moreover, MBZ impacts primary cilia biology and DNA damage responses [52], and can modulate multiple cancer-related signaling pathways [53]. These reports collectively underscore the pleiotropic and multi-target nature of MBZ’s anticancer activity. However, our study provides convergent and consistent experimental evidence supporting that MDZ exerts its antitumor effects through modulation of the USP5-IMPDH2 pathway. Therefore, the USP5-IMPDH2 axis may represent an important molecular mechanism mediating the therapeutic efficacy of MDZ in ESCC.

This study revealed a molecular mechanism by which USP5 stabilizes IMPDH2 through deubiquitination, thereby enhancing purine metabolism and promoting the progression of ESCC. These findings broadened our understanding of cancer metabolic reprogramming, emphasizing the critical role of nucleotide metabolism in tumor cell proliferation. Furthermore, we demonstrated that the USP5 inhibitor Mebendazole not only significantly suppressesed ESCC cell growth but also enhanced their sensitivity to Oxaliplatin, highlighting its potential for drug repurposing in cancer therapy (Fig. 8H). Collectively, this work identified a novel metabolic target for therapeutic intervention and proposed a new strategy to address variability in Oxaliplatin response, establishing a key link between metabolic regulation and chemotherapeutic efficacy.

## Supplementary information

supplementary figure1

supplementary figure2

supplementary figure3

supplementary figure4

supplementary figure5

supplementary figure6

supplement figure legend

original WB band

## Data Availability

Data supporting the findings of this study are available within the article and its supplementary information files. Additional data are available from the corresponding author upon reasonable request.
