# Supplementary figures and images for: USP5 regulates purine metabolism and represents a therapeutic target in esophageal cancer
Source: Cell Death Dis. 2026 Apr 1;17(1):439. doi: 10.1038/s41419-026-08683-4 (PMC13168492; doi:10.1038/s41419-026-08683-4)

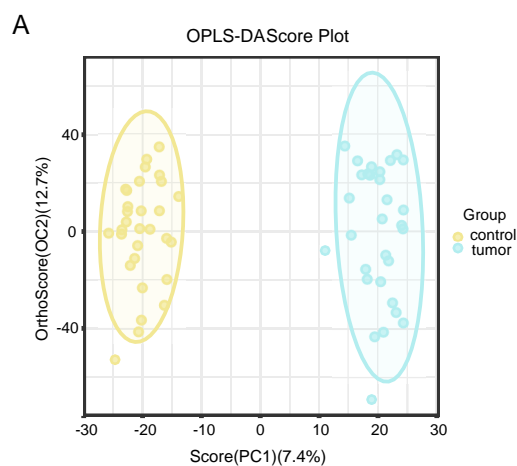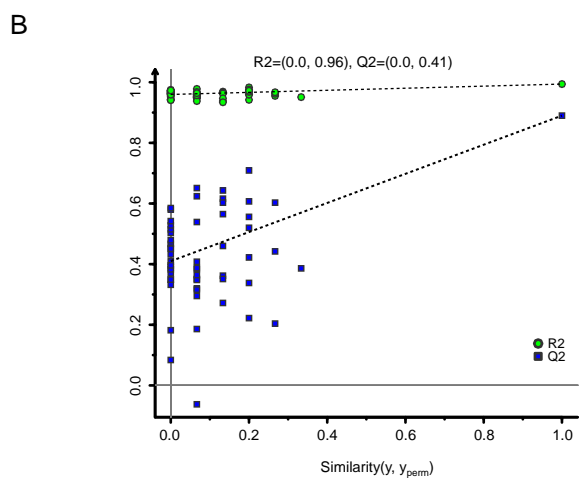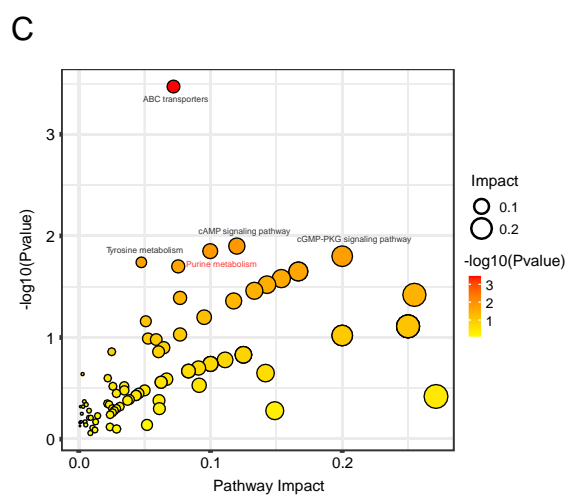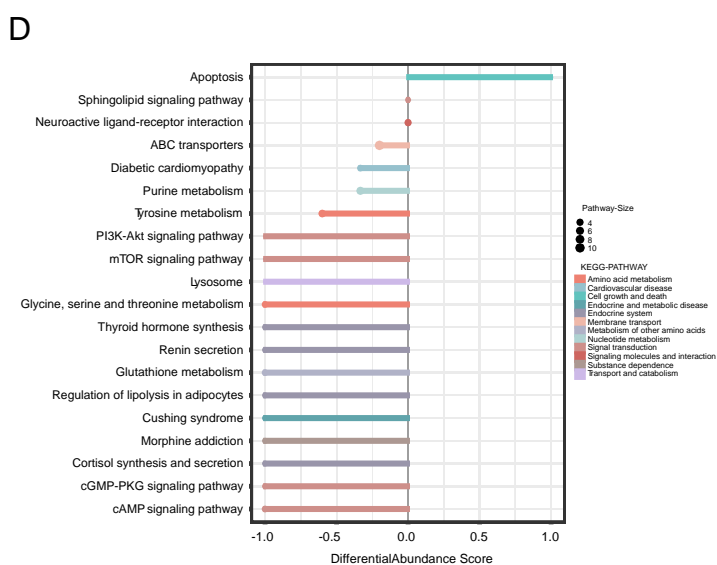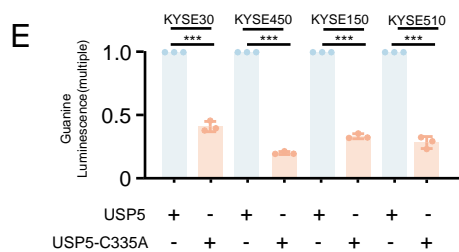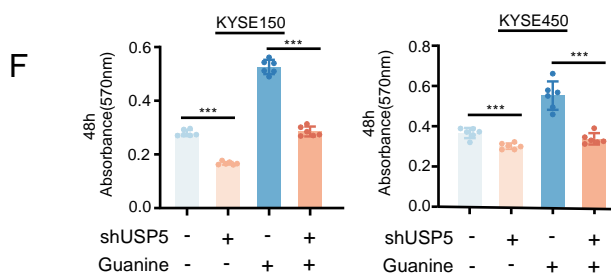

Supplement: Supplementary file 1 — supplementary figure1 [file 41419_2026_8683_MOESM1_ESM.pdf]

A

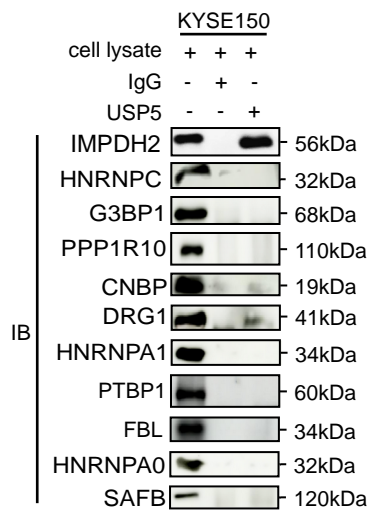

B

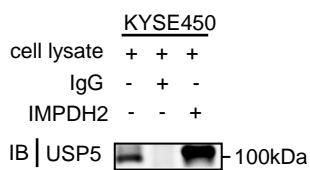

C

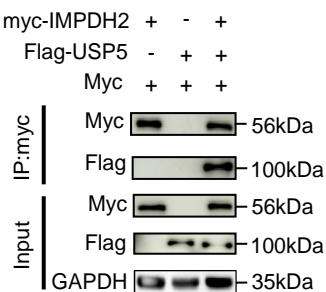

D

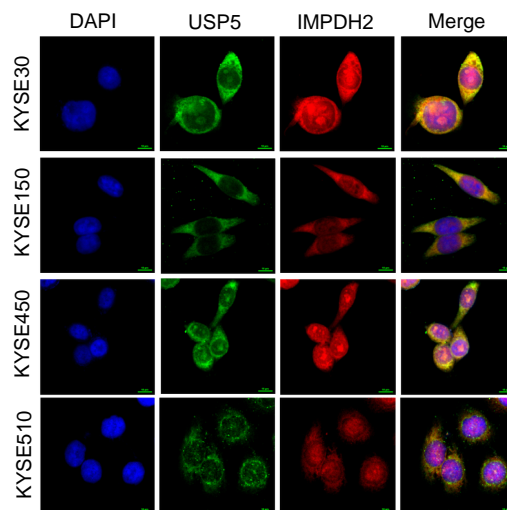

E

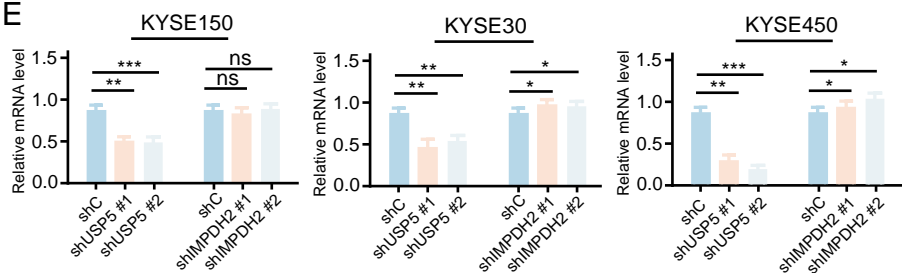

F

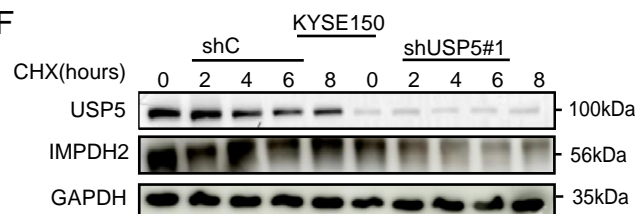

G

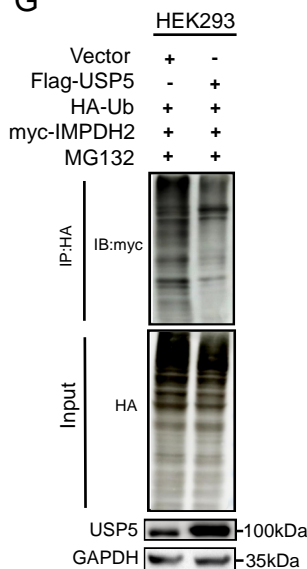

H

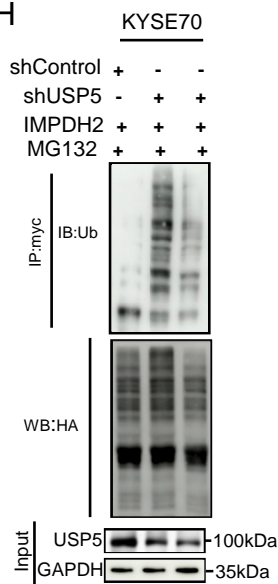

KYSE150

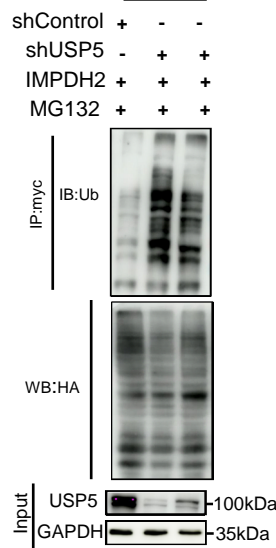

I

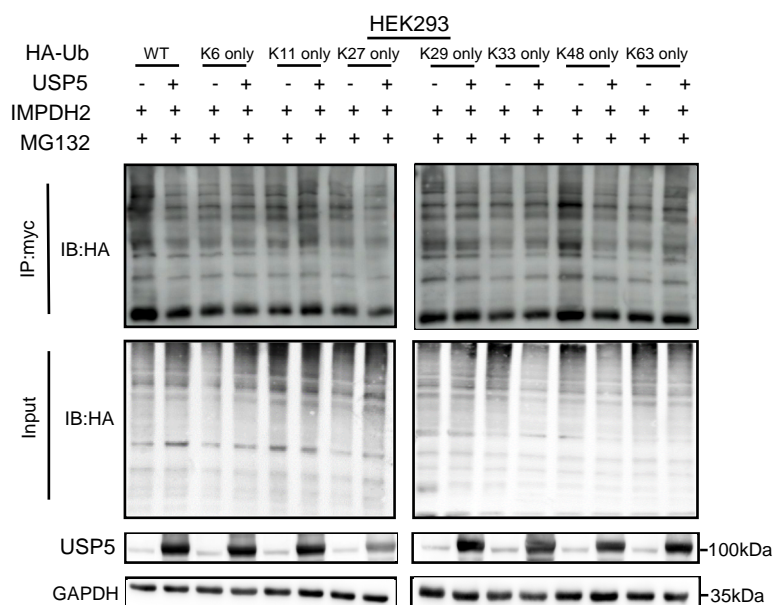

J

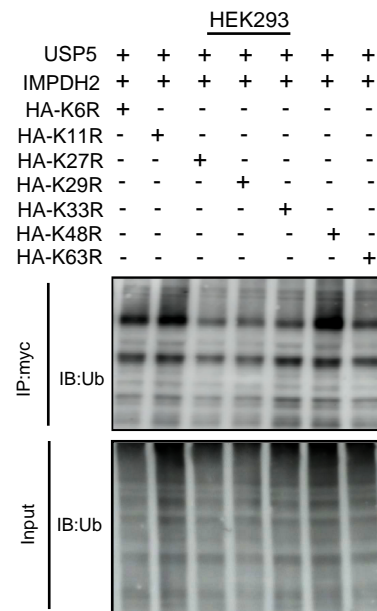

Supplement: Supplementary file 2 — supplementary figure2 [file 41419_2026_8683_MOESM2_ESM.pdf]

A

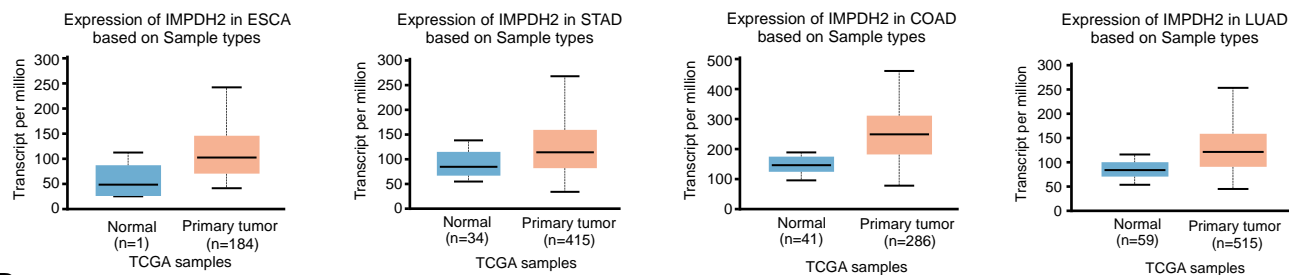

B

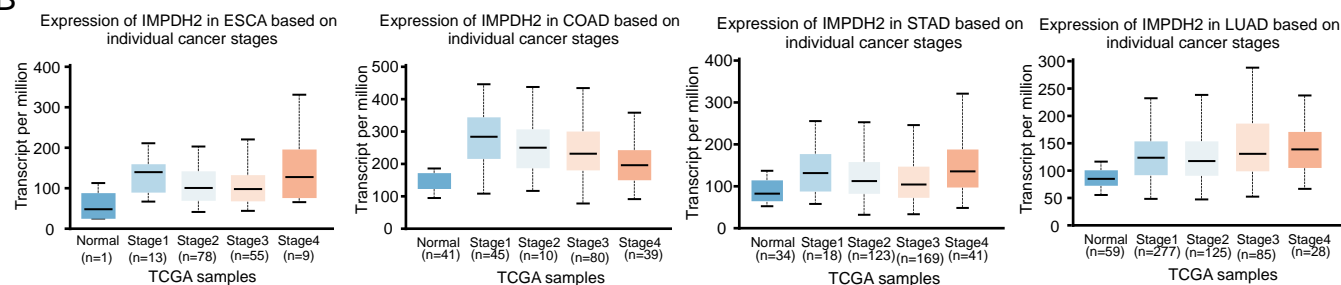

C

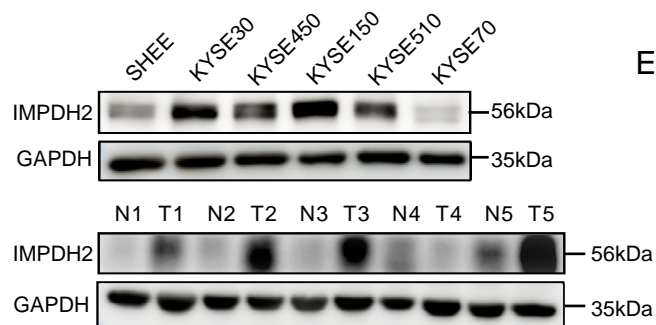

E

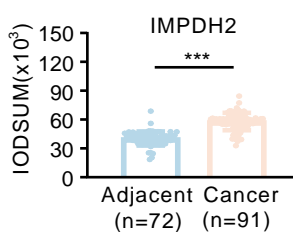

F

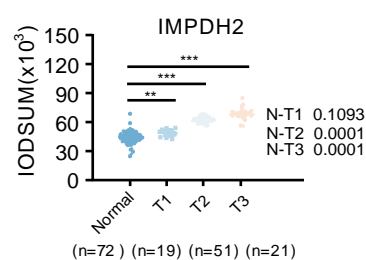

D

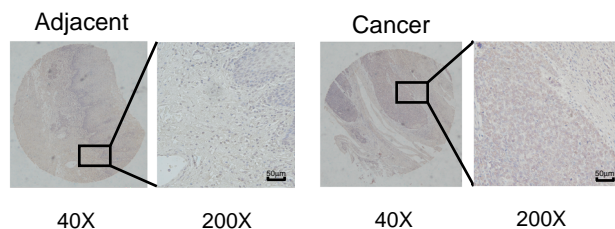

G

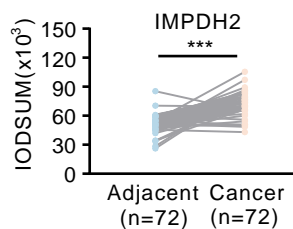

H

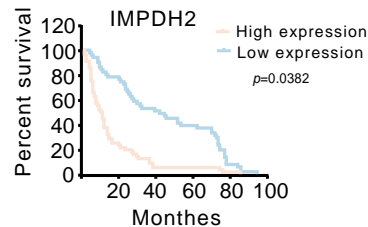

Supplement: Supplementary file 3 — supplementary figure3 [file 41419_2026_8683_MOESM3_ESM.pdf]

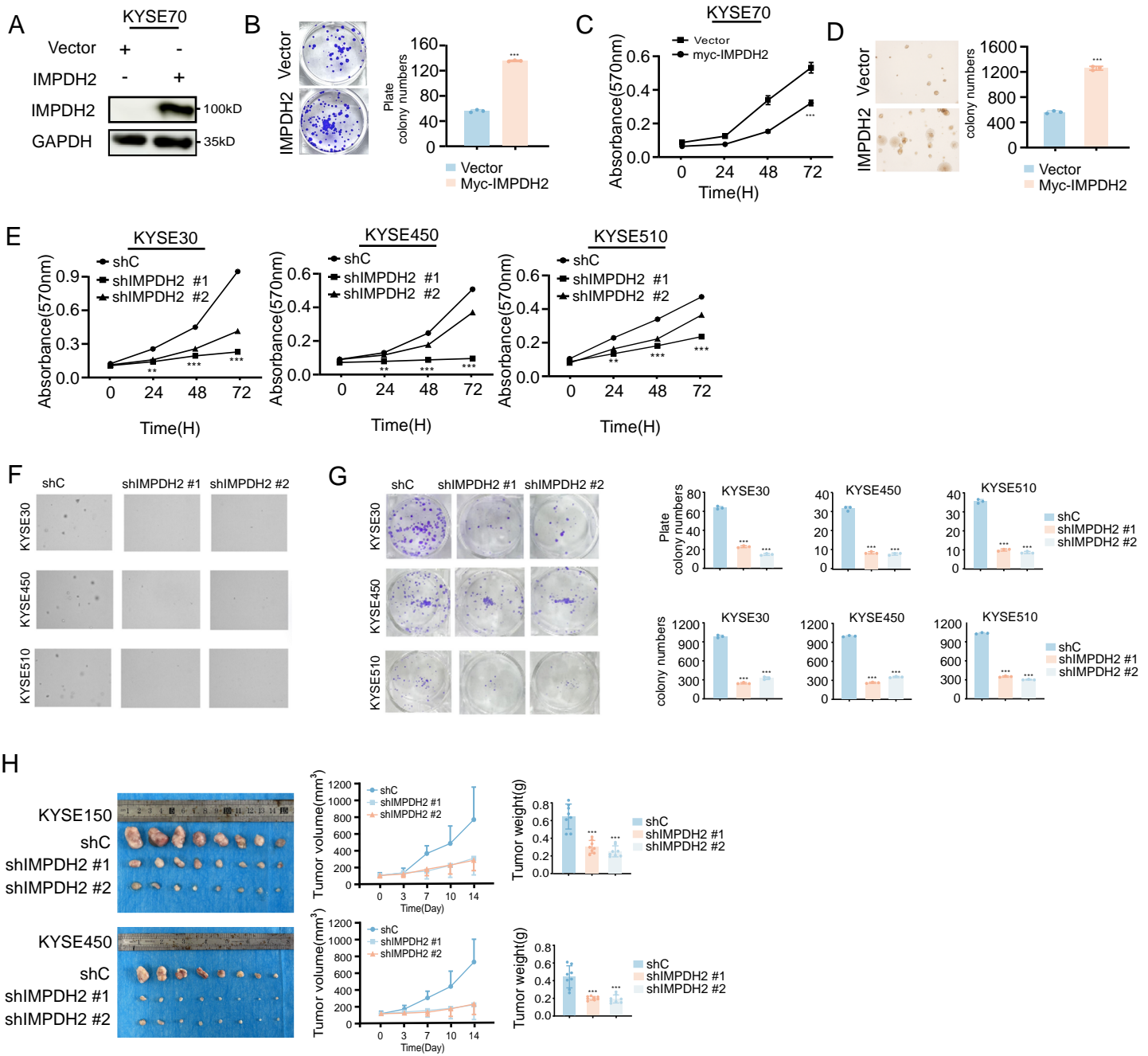

Supplement: Supplementary file 4 — supplementary figure4 [file 41419_2026_8683_MOESM4_ESM.pdf]

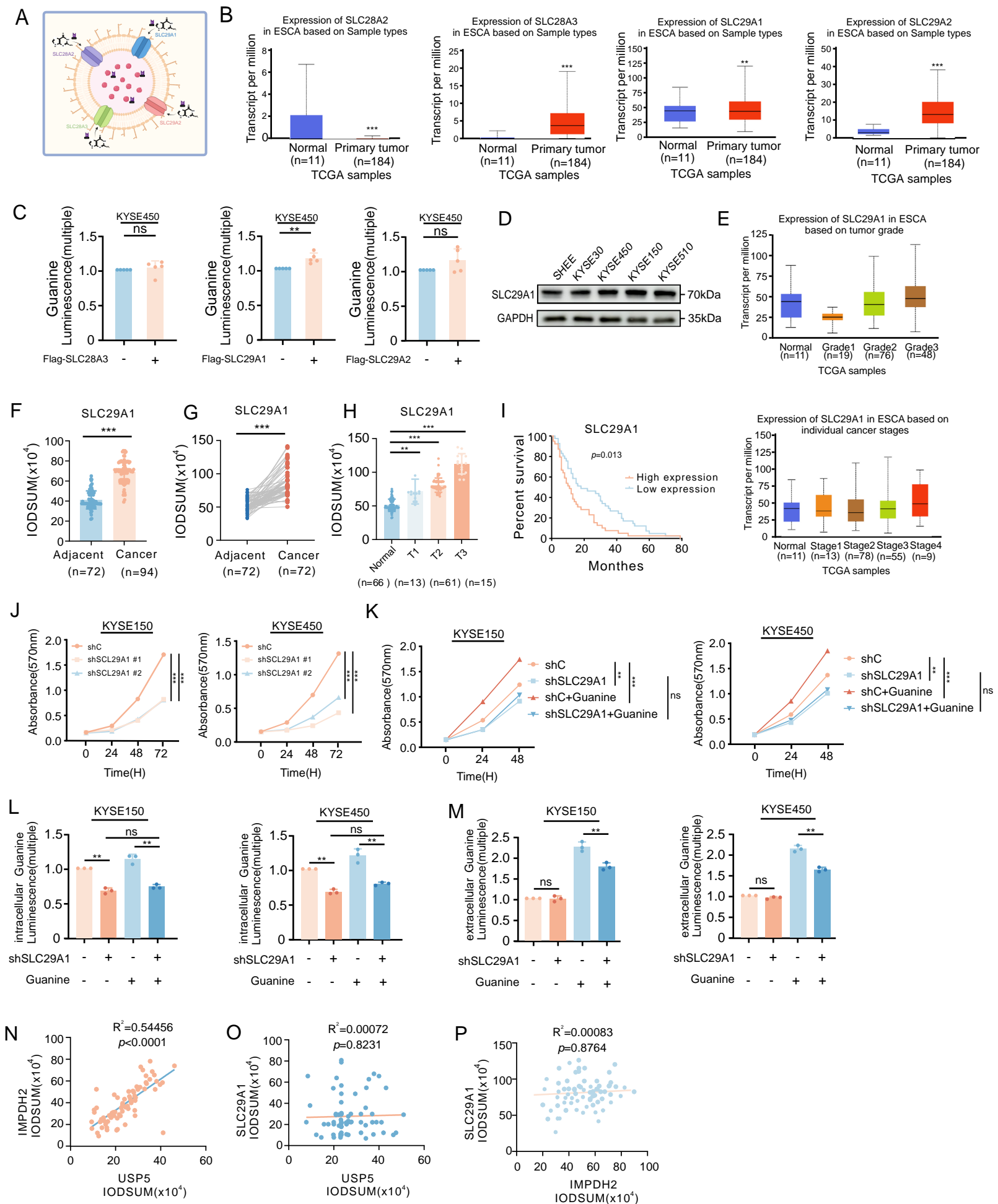

Supplement: Supplementary file 5 — supplementary figure5 [file 41419_2026_8683_MOESM5_ESM.pdf]

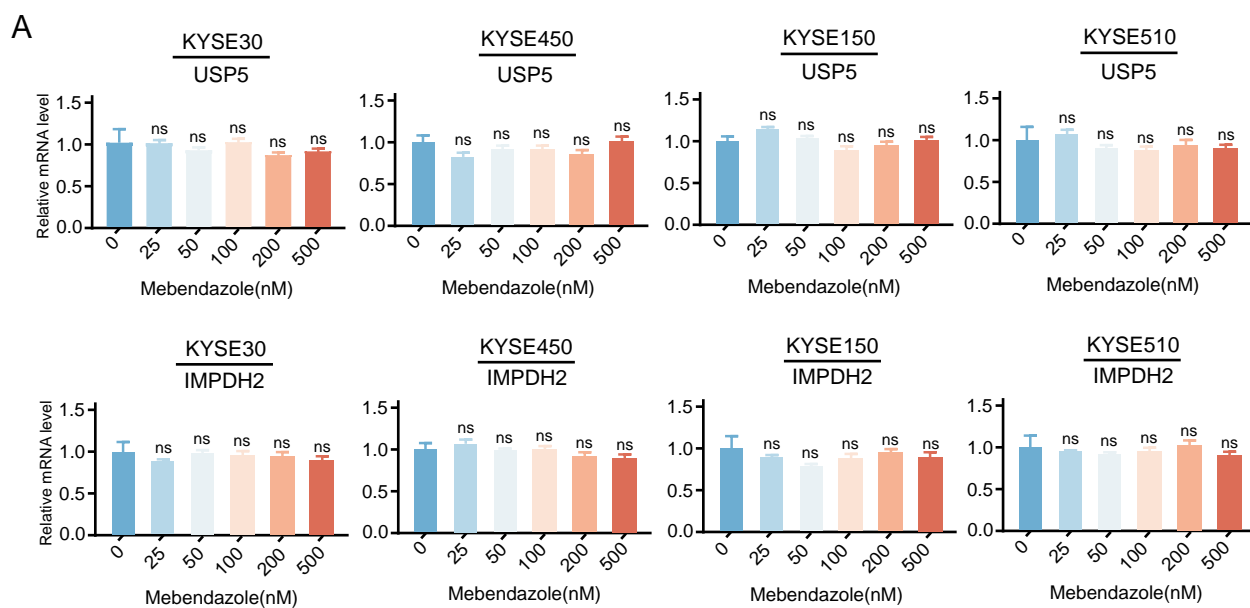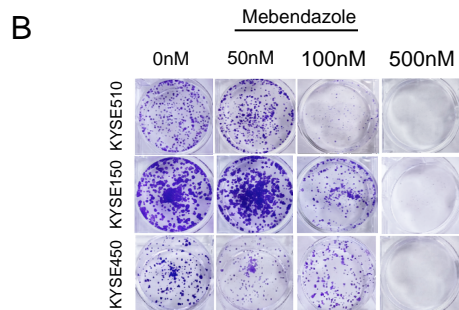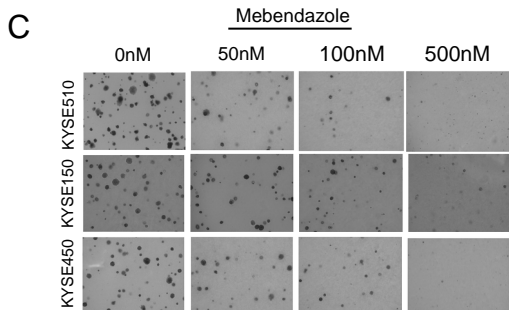

Supplement: Supplementary file 6 — supplementary figure6 [file 41419_2026_8683_MOESM6_ESM.pdf]
