# Supplementary material for: USP5 regulates purine metabolism and represents a therapeutic target in esophageal cancer
Source: Cell Death Dis. 2026 Apr 1;17(1):439. doi: 10.1038/s41419-026-08683-4 (PMC13168492; doi:10.1038/s41419-026-08683-4)
Supplement: Supplementary file 7 — supplement figure legend [file 41419_2026_8683_MOESM7_ESM.docx]

**Supplementary Figure 1. Validation of metabolomic profiling and functional importance of purine biosynthesis in ESCC.**

1. OPLS-DA score plot showing ESCC tumor tissues and adjacent normal tissues.
   **B.** Volcano plot displaying significantly different metabolites between tumor and normal tissues.
   **C–D.** KEGG pathway enrichment analysis showing significant enrichment in ESCC.
   **E.** In ESCC cells, Flag-USP5-WT and Flag-USP5-C335A plasmids were transfected in KYSE30, KYSE450, KYSE150 and KYSE510, and guanine levels were measured.
   **F.** In KYSE150 and KYSE450 cells with USP5 knockdown, exogenous guanine was added, and guanine levels were measured.

Data are presented as mean ± SD. Statistical significance was assessed by one-way ANOVA (E, F) as appropriate. **p* < 0.05; ***p* < 0.01; ****p* < 0.001.

**Supplementary Figure 2. USP5 mediates the deubiquitination of IMPDH2 at the K489 site through K48-linked ubiquitin chains.**

1. Endogenous co-IP confirmed the interaction between USP5 and HNRNPC, G3BP1, PPP1R10, CNBP, DRG1, HNRNPA1, PTBP1, FBL, HNRNPA0, SAFB.
2. Endogenous co-IP confirmed the interaction between USP5 and IMPDH2.
3. Exogenous co-IP confirmed the interaction between USP5 and IMPDH2.
4. IF staining showed co-localization of USP5 (green) and IMPDH2 (red) in the cytoplasm of ESCC cells (blue represents DAPI staining).
5. IMPDH2 mRNA levels after USP5 knockdown (shUSP5) were detected by qRT-PCR.
6. IMPDH2 protein stability was assessed by WB after USP5 knockdown, and CHX treatment at various time points.
7. Flag-USP5 plasmids were transfected into HEK293 cells, and IMPDH2 ubiquitination levels were assessed by Western blot after MG132 treatment.
8. In USP5-knockdown KYSE150 and KYSE70 cells, IMPDH2 ubiquitination levels were assessed by Western blot following MG132 treatment.

**I.** In HEK293 cells, HA-Ub, HA-K6, HA-K11, HA-K27, HA-K29, HA-K33, HA-K48, and HA-K63 ubiquitin chain plasmids were transfected, followed by MG132 treatment, and IMPDH2 ubiquitination levels were assessed by Western blot.

**J.** In HEK293 cells, HA-Ub, HA-K6R, HA-K11R, HA-K27R, HA-K29R, HA-K33R, HA-K48R, and HA-K63R ubiquitin chain plasmids were transfected, followed by MG132 treatment, and IMPDH2 ubiquitination levels were assessed by Western blot.

Data are presented as mean ± SD. Statistical significance was assessed by one-way ANOVA (E) as appropriate. **p* < 0.05; ***p* < 0.01; ****p* < 0.001.

**Supplementary Figure 3. IMPDH2 is aberrantly upregulated in ESCC and is associated with disease progression.**

**A.** Pan-cancer analysis of TCGA data showing IMPDH2 expression across multiple cancer types.

**B.** Correlation between IMPDH2 expression levels and clinical stage progression in ESCC patients.

**C.** Western blot analysis of IMPDH2 protein levels in ESCC cell lines and primary tumor tissues.

**D.** Immunohistochemistry (IHC) analysis of IMPDH2 expression in tumor tissues using a commercial ESCC tissue microarray (n = 66 pairs).

**E-F**. Quantification of IMPDH2 protein levels by IHC in ESCC tissues and adjacent normal tissues, presented as mean IOD (integrated optical density).

**G.** IHC analysis of IMPDH2 expression across different tumor stages (T1–T3) in ESCC tissues.

**H**. Kaplan–Meier survival curve analysis of the correlation between IMPDH2 expression and overall survival in ESCC patients.

Data are presented as mean ± SD. Statistical significance was assessed by paired two-tailed Student’s t-test（A, G）, unpaired two-tailed Student’s t-test（E）, log-rank test (H) or one-way ANOVA (B, F) as appropriate3. **p* < 0.05; ***p* < 0.01; ****p* < 0.001.

**Supplementary Figure 4. IMPDH2 promotes ESCC cell proliferation and tumor growth in vitro and in vivo.**

**A–D.** Overexpression of IMPDH2 in KYSE410 cells assessed by cell proliferation (MTT assay) and colony formation ability.

**E.** MTT assay assessing the effect of IMPDH2 knockdown on cell proliferation in KYSE30, KYSE450, and KYSE510 cells.

**F–G.** Foci formation assay and soft agar assay evaluating the impact of IMPDH2 knockdown on ESCC cell colony formation ability.

**H.** In vivo CDX mouse models using KYSE150 and KYSE450 cells, with tumor volume and weight quantified.

Data are presented as mean ± SD. Statistical significance was assessed by paired two-tailed Student’s t-test(A, D), unpaired two-tailed Student’s t-test（A, C）two-way ANOVA (C, E, H) or one-way ANOVA (F, G, H) as appropriate. **p* < 0.05; ***p* < 0.01; ****p* < 0.001.

**Supplementary Figure 5. SLC29A1 functions as a guanine transporter to promote guanine uptake and ESCC progression.**
**A**. Schematic of purine transporters potentially involved in guanine transport.
**B**. Expression levels of SLC28A2, SLC28A3, SLC29A1, and SLC29A2 in ESCC were analyzed using the TCGA database.

**C**. Guanine levels in KYSE450 cells after overexpression of SLC28A3, SLC29A1, and SLC29A2.

**D**. Western blot analysis of SLC29A1 expression in ESCC cell lines.

**E.** Association of SLC29A1 with pathological grade and clinical stage of ESCC using TCGA dataset analysis.

**F-G.** Quantification of IMPDH2 protein levels by IHC in ESCC tissues and adjacent normal tissues, presented as mean IOD (integrated optical density).

**H**. IHC analysis of IMPDH2 expression across different tumor stages (T1–T3) in ESCC tissues.

**I**. Kaplan–Meier survival curve analysis of the correlation between IMPDH2 expression and overall survival in ESCC patients.

**J**. MTT assay of KYSE150 cells upon SLC29A1 knockdown in KYSE150 and KYSE450.

**K.** MTT assay of the effect of exogenous guanine supplementation on cell viability upon SLC29A1 knockdown in KYSE150 and KYSE450 cells..

**L-M**. Quantification of intracellular and extracellular guanine levels following SLC29A1 knockdown with or without exogenous guanine supplementation in KYSE150 and KYSE450.
**N.** Correlation between USP5 and SLC29A1 expression in ESCC tissues.

**O.** Correlation between USP5 and IMPDH2 expression in ESCC tissues.

**P.** Correlation between IMPDH2 and SLC29A1 expression in ESCC tissues.

Data are presented as mean ± SD. Statistical significance was assessed by paired two-tailed Student’s t-test（B, G）, unpaired two-tailed Student’s t-test（C, F）, log-rank test (I) two-way ANOVA (J, K) or one-way ANOVA (H, L, M) as appropriate. **p* < 0.05; ***p* < 0.01; ****p* < 0.001.

**Supplementary Figure 6.** **MDZ downregulates USP5 and inhibits ESCC cell growth.**

**A.** qPCR analysis of USP5 and IMPDH2 mRNA expression levels following MDZ treatment.
**B–C.** Foci formation and soft agar assays assessing the effect of MDZ treatment on the colony formation ability of KYSE510, KYSE150, and KYSE450 cells.

Data are presented as mean ± SD. Statistical significance was assessed by one-way ANOVA (A) as appropriate. **p* < 0.05; ***p* < 0.01; ****p* < 0.001.
