# Supplementary material for: USP5 regulates purine metabolism and represents a therapeutic target in esophageal cancer
Source: Cell Death Dis. 2026 Apr 1;17(1):439. doi: 10.1038/s41419-026-08683-4 (PMC13168492; doi:10.1038/s41419-026-08683-4)

Figure 1J-USP5

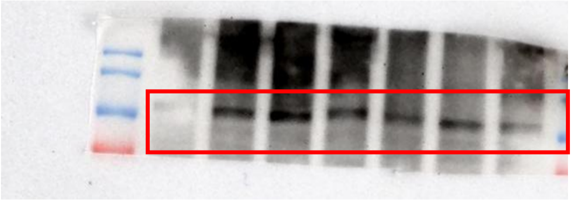

Figure 1K-USP5

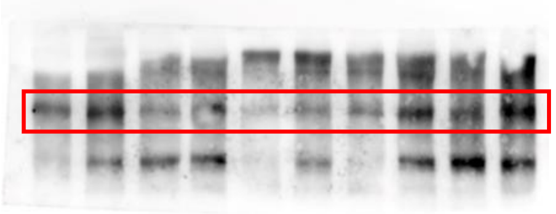

Figure 1K-GAPDH

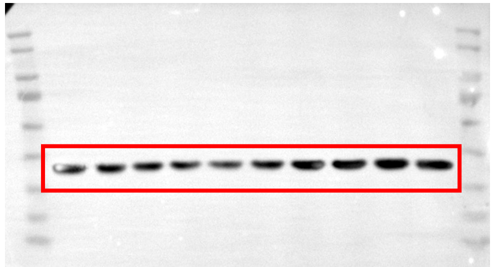

Figure 1J-GAPDH

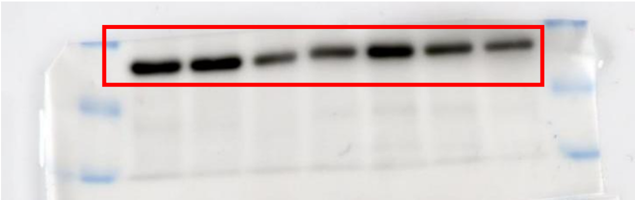

Figure2A-USP5-KYSE30

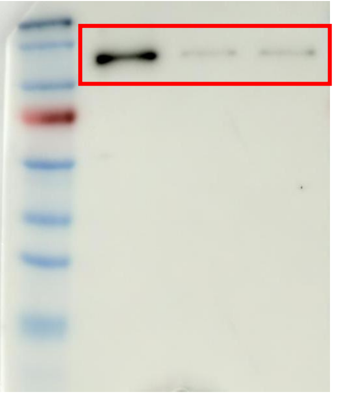

Figure2A-GAPDH-KYSE30

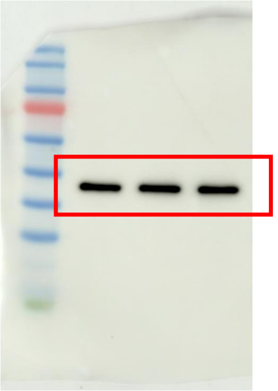

Figure2A-USP5-KYSE450

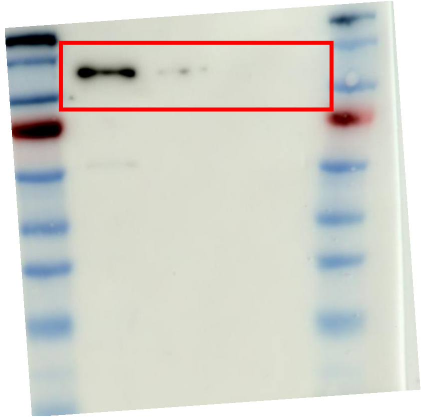

Figure2A-GAPDH-KYSE450

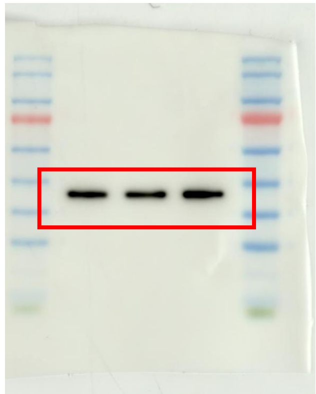

Figure2A-USP5-KYSE150

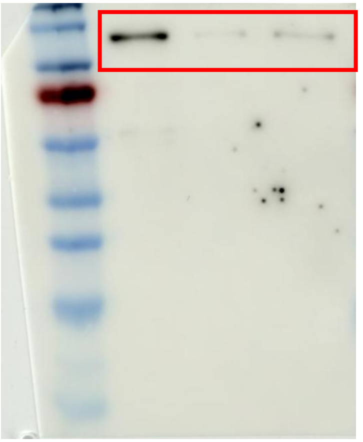

Figure2A-GAPDH-KYSE150

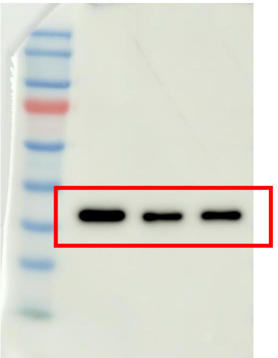

Figure2A-USP5-KYSE510

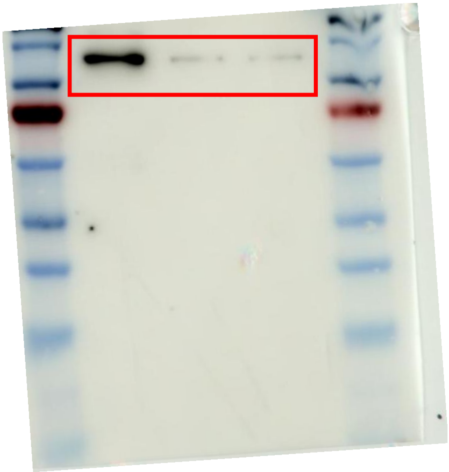

Figure2A-GAPDH-KYSE510

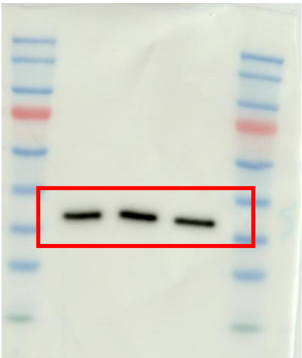

Figure2E-USP5-KYSE70

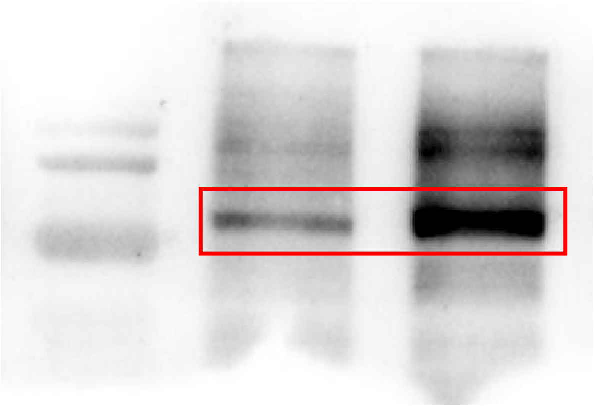

Figure2E-GAPDH-KYSE70

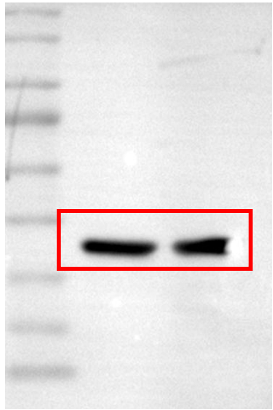

Figure4B-IMPDH2-150

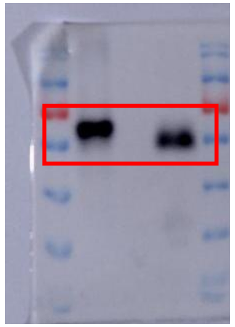

Figure4B-USP5-30

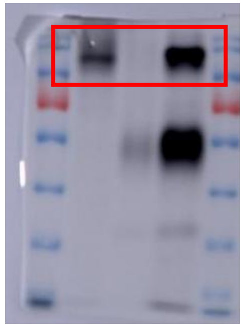

Figure4C-IP:FLAG-FLAG

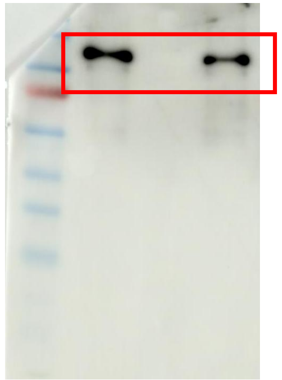

Figure4C-IP:FLAG-MYC

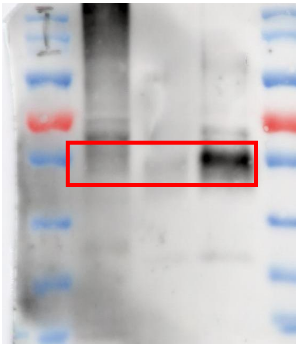

Figure4D-IP:FLAG-INPUT-FLAG

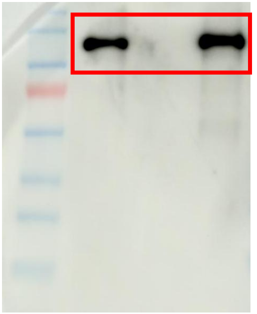

Figure4D-IP:FLAG-INPUT-MYC

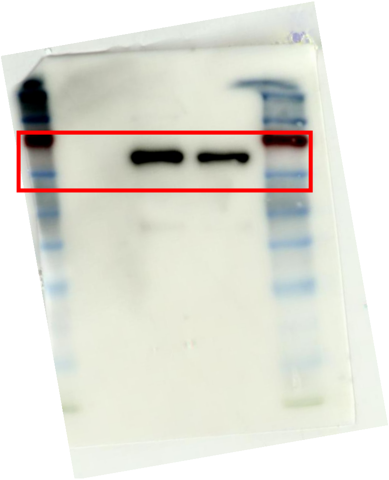

Figure4D-IP:FLAG-GAPDH

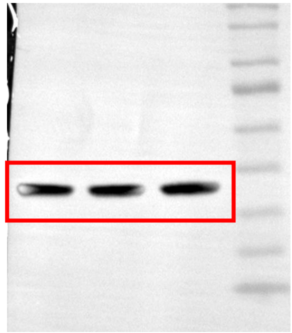

Figure4E-USP5-KYSE30

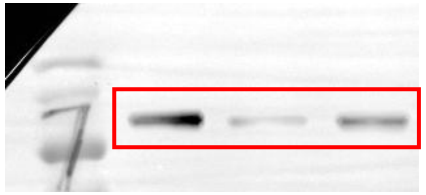

Figure4E-USP5-KYSE450

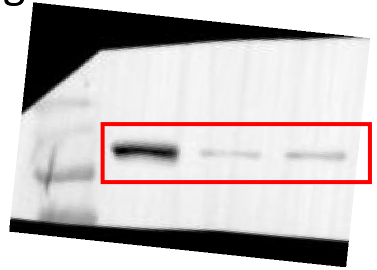

Figure4E-USP5-KYSE150

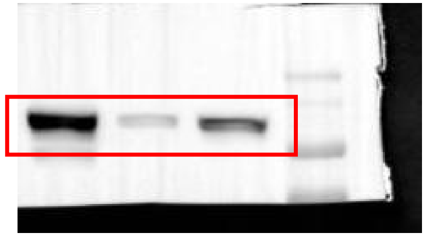

Figure4E-USP5-KYSE510

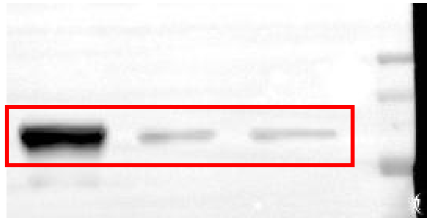

Figure4E-IMPDH2-KYSE30

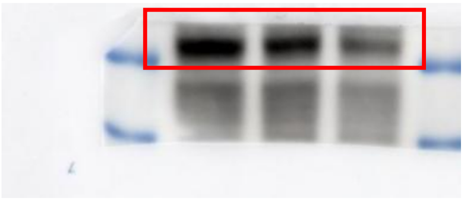

Figure4E-IMPDH2-KYSE450

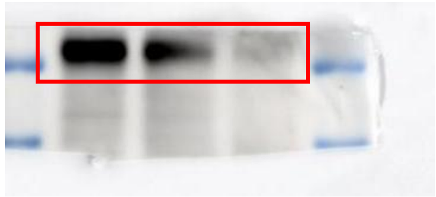

Figure4E-IMPDH2-KYSE150

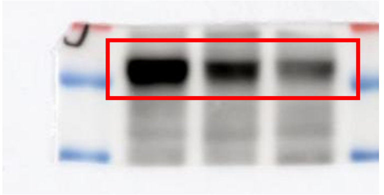

Figure4E-IMPDH2-KYSE510

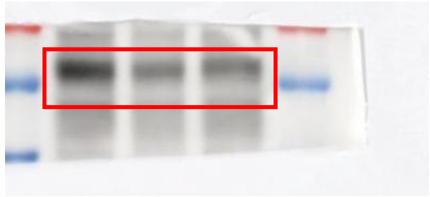

Figure4E-GAPDH-KYSE30

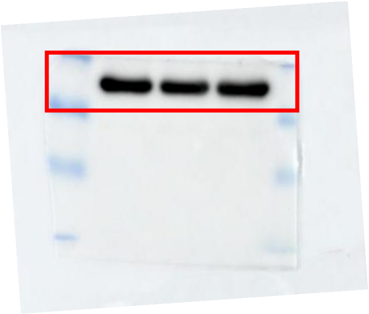

Figure4E-GAPDH-KYSE450

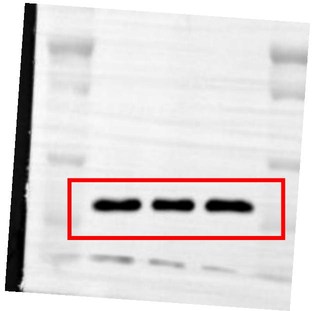

Figure4E-GAPDH-KYSE150

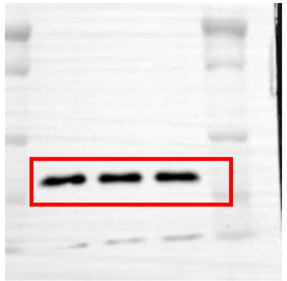

Figure4E-GAPDH-KYSE510

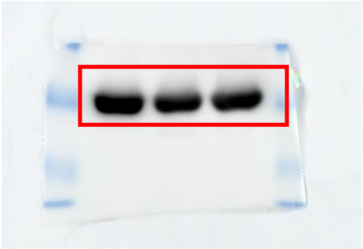

Figure4F-USP5-KYSE70

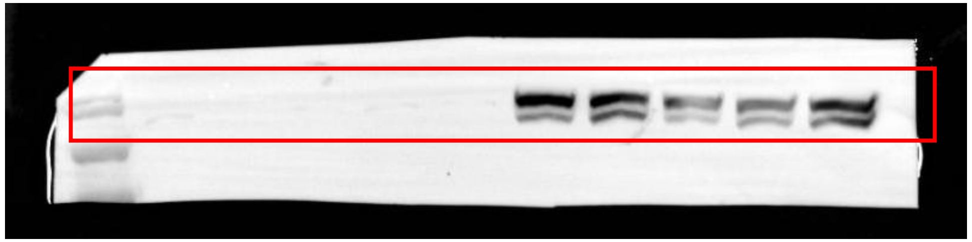

Figure4F-IMPDH2-KYSE70

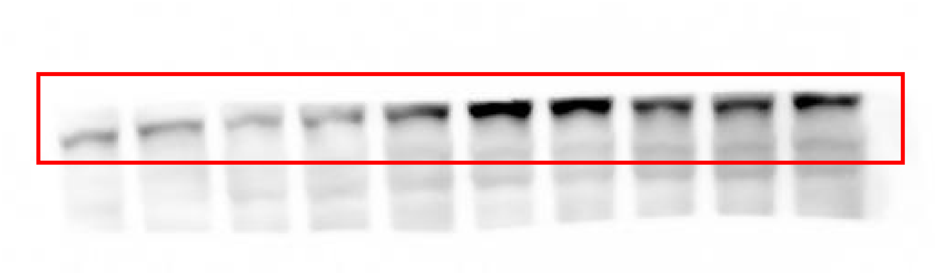

Figure4F-GAPDH-KYSE70

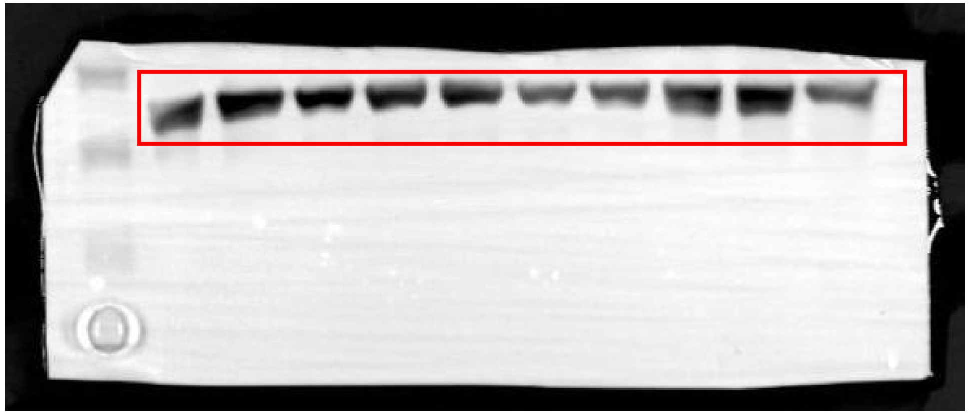

Figure4G-IMPDH2-3MA

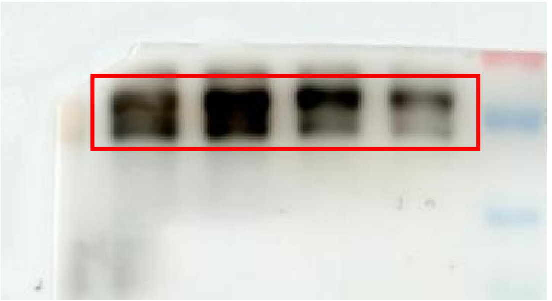

Figure4G-IMPDH2-MG132

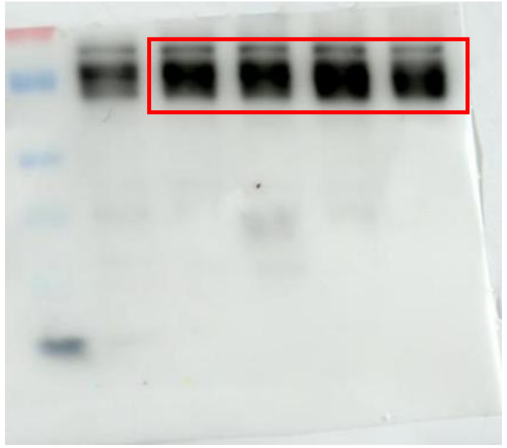

Figure4G-GAPDH-3MA

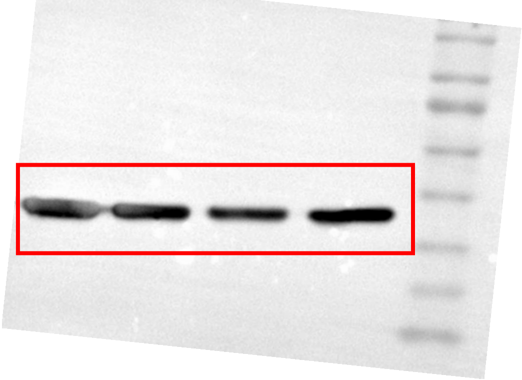

Figure4G-GAPDH-MG132

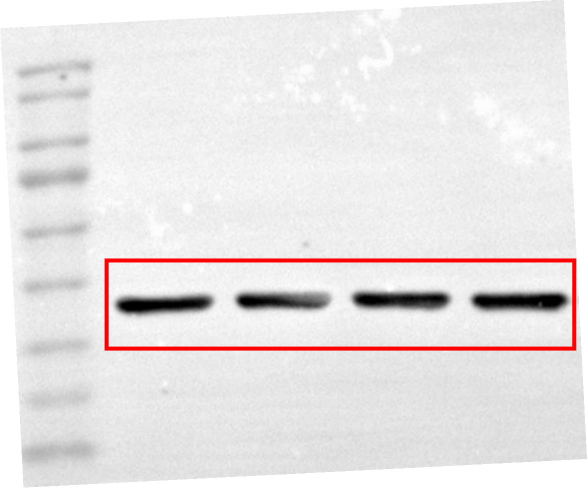

Figure4H-Ub

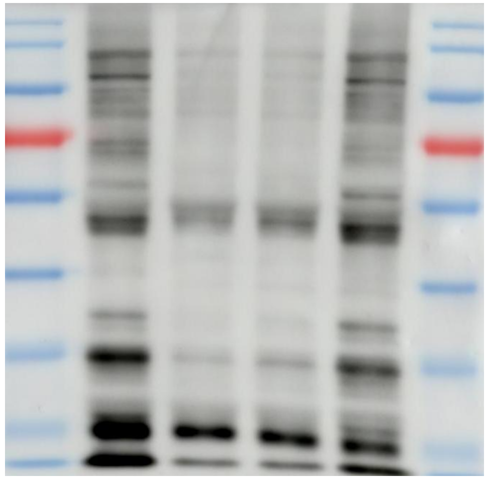

Figure4H-IMPDH2

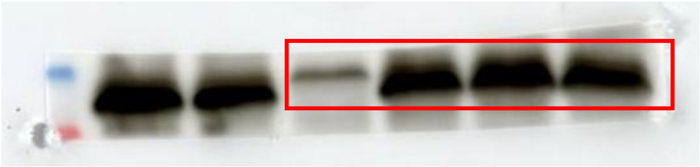

Figure4H-USP5

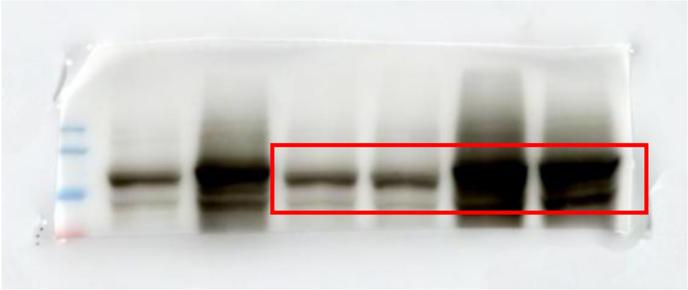

Figure4H-GAPDH

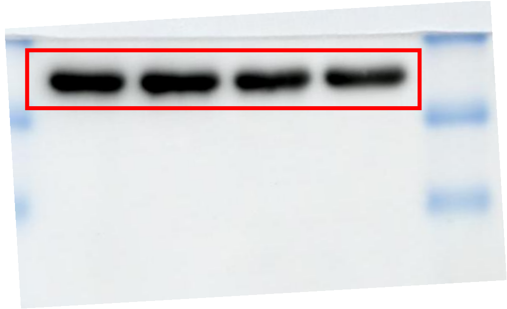

Figure4I-Ub

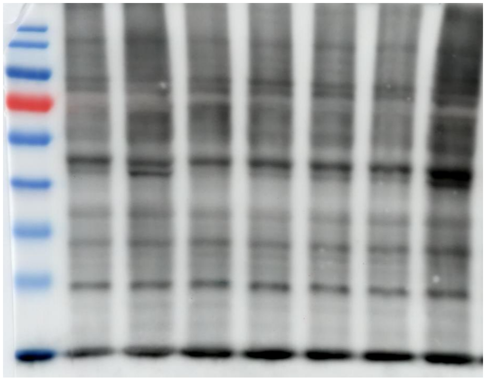

Figure4I-INPUT-Ub

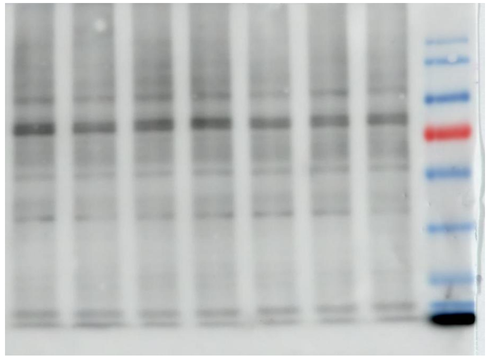

Figure4J-SYVN1-KYSE150

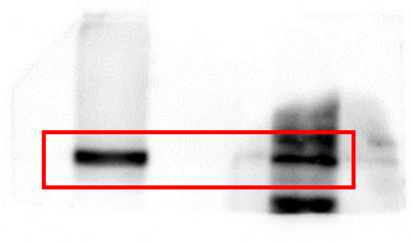

Figure4J-SYVN1-KYSE450

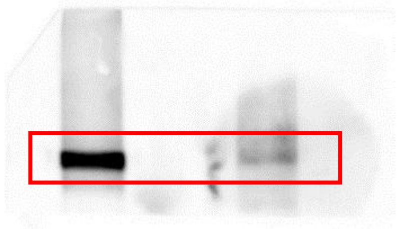

Figure4J-IMPDH2-KYSE150

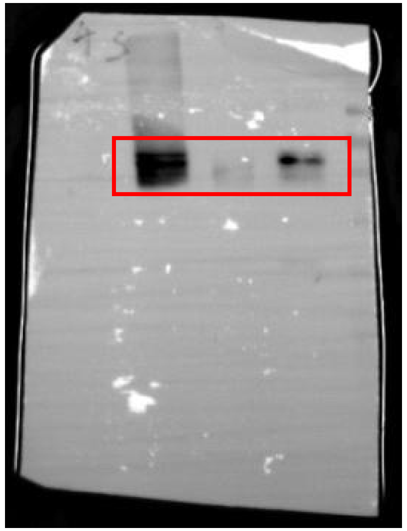

Figure4K-HA-HEK293

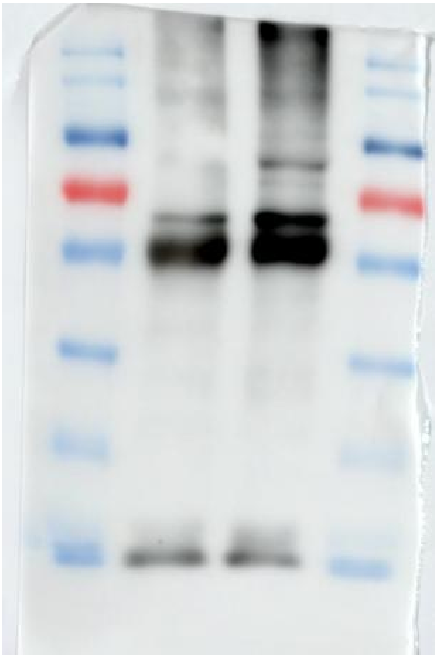

Figure4K-GAPDH

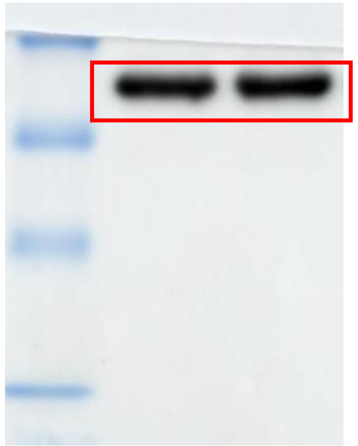

Figure5K-IMPDH2

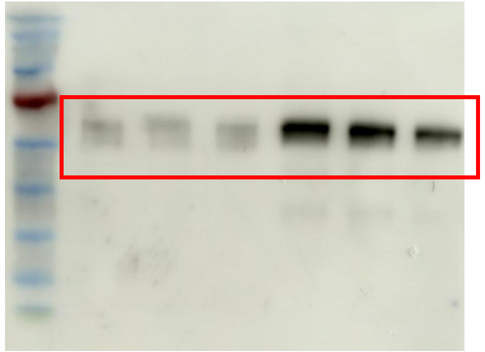

Figure5K-GAPDH

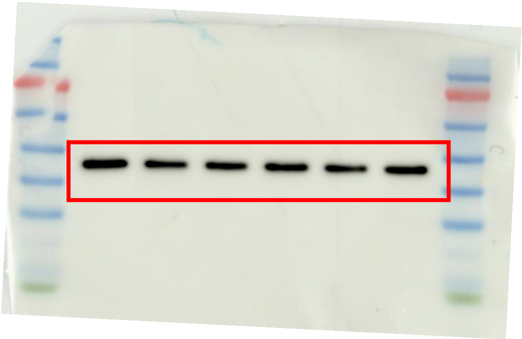

Figure5K-USP5

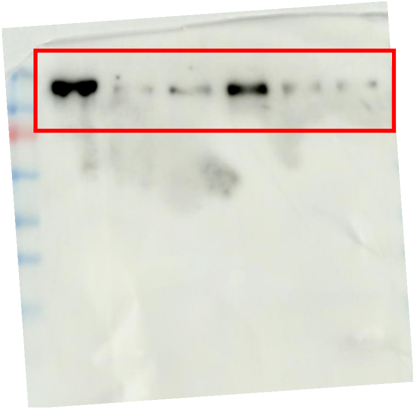

Figure6A-USP5-KYSE30

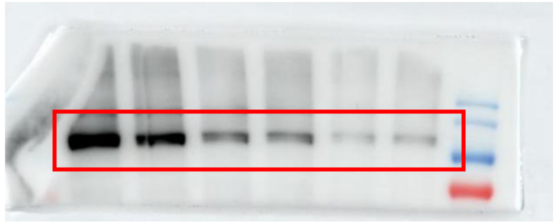

Figure6A-USP5-KYSE450

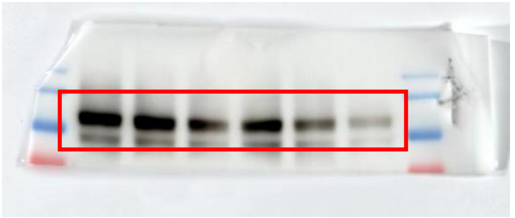

Figure6A-IMPDH2-KYSE30

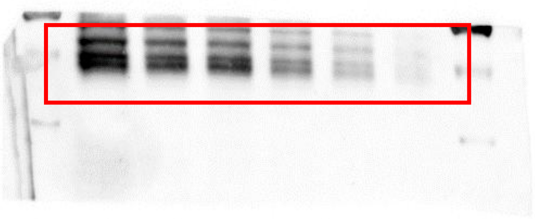

Figure6A-IMPDH2-KYSE450

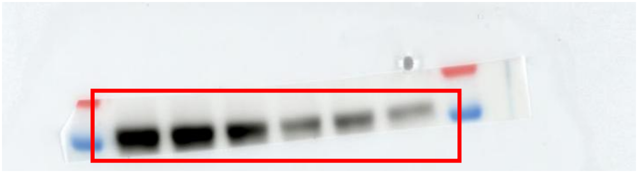

Figure6A-GAPDH-KYSE30

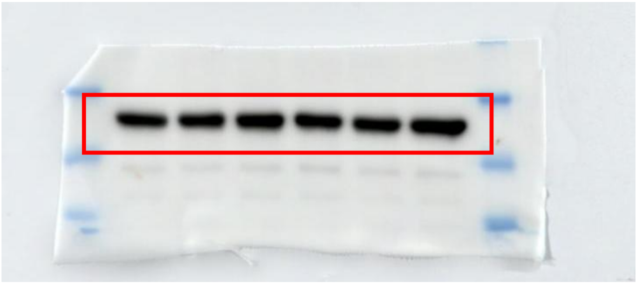

Figure6A-GAPDH-KYSE450

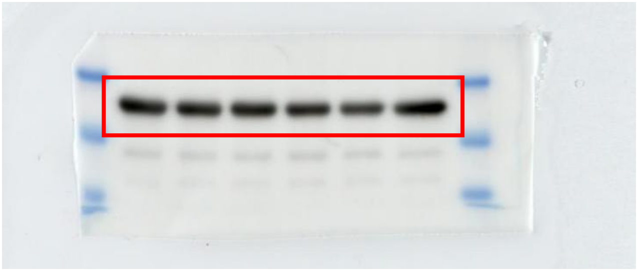

Figure6C-USP5-KYSE150

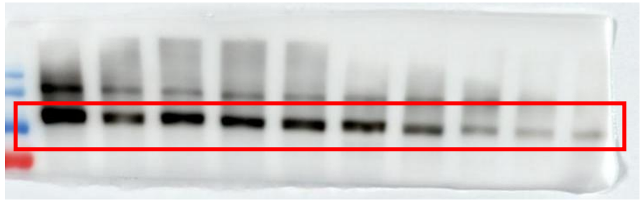

Figure6C-USP5-KYSE450

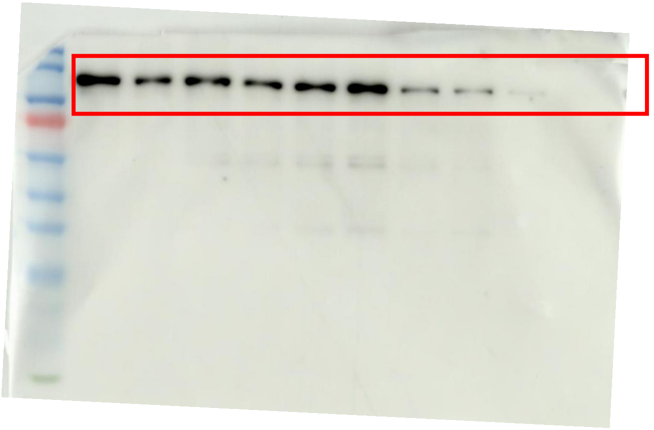

Figure6C-GAPDH-KYSE150

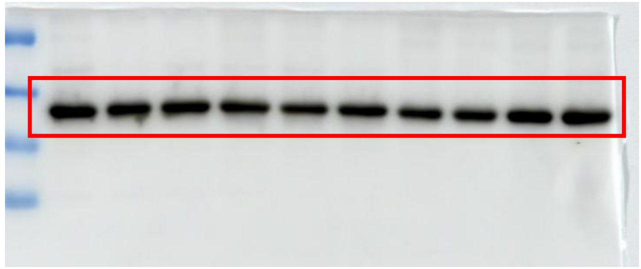

Figure6C-GAPDH-KYSE450

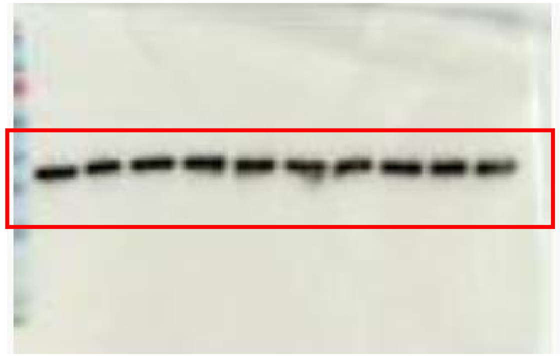

Figure6D-Ub-KYSE70

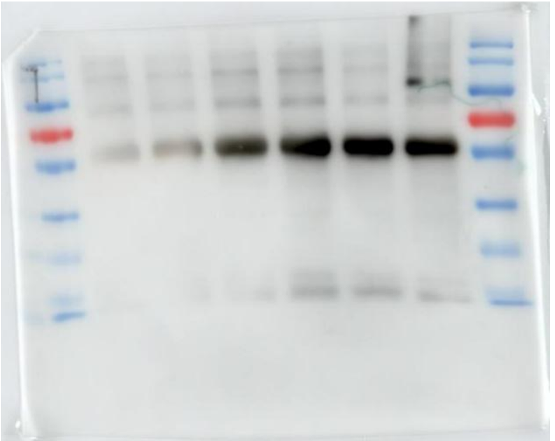

Figure6D-Ub-KYSE30

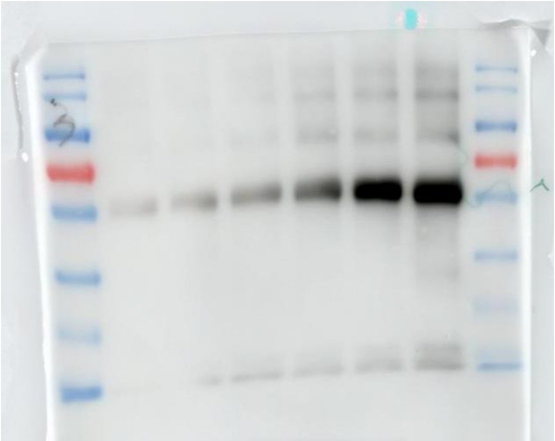

Figure6E-Ub-KYSE30

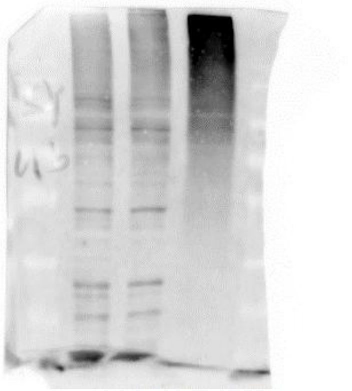

Figure6E-Ub-KYSE450

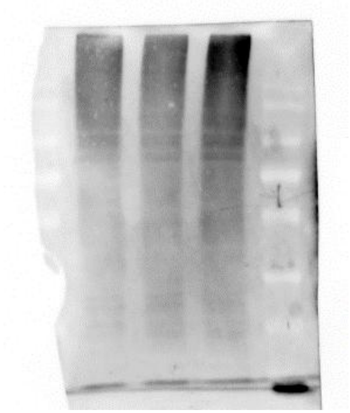

Figure6E-SYVN1-KYSE30

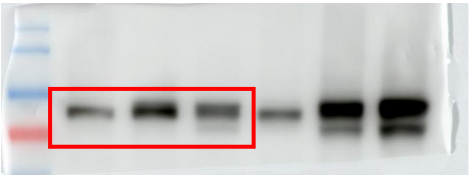

Figure6E-SYVN1-KYSE450

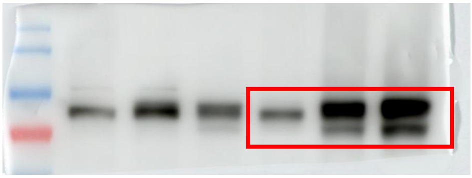

Figure6E-GAPDH-KYSE30

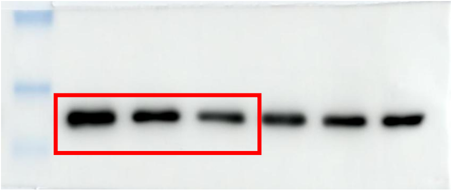

Figure6E-GAPDH-KYSE450

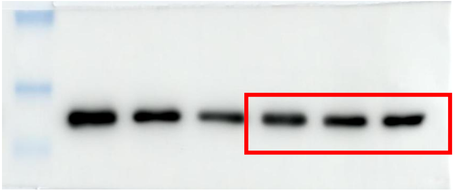

FigureS2A-IMPDH2

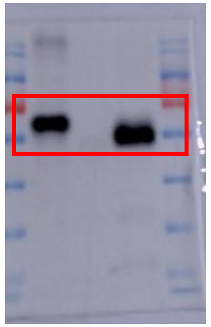

FigureS2A-HNRNPC

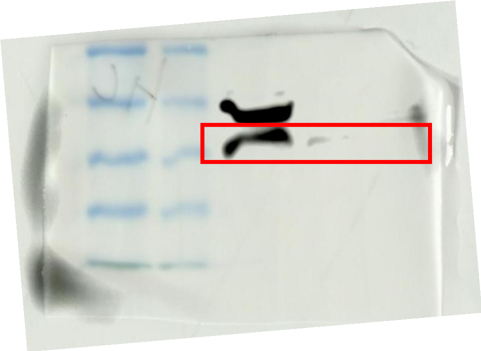

FigureS2A-G3BP1

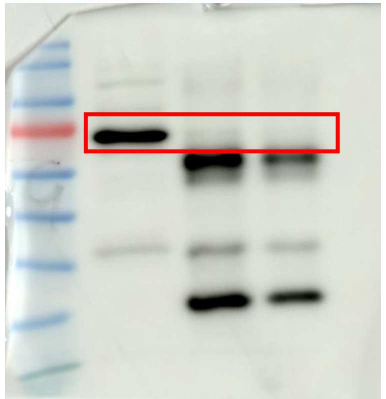

FigureS2A-PPP1R10

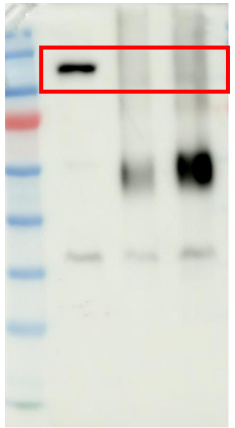

FigureS2A-CNBP

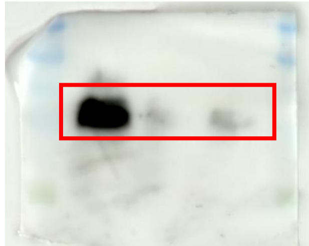

FigureS2A-DRG1

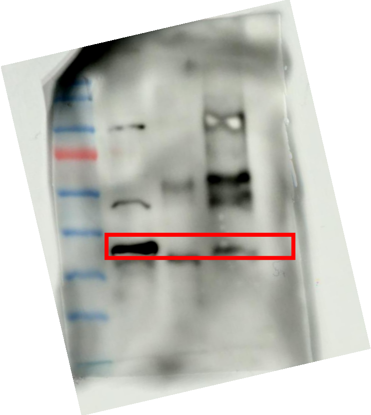

FigureS2A-HNRNPA1

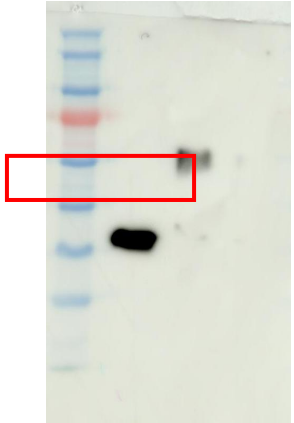

FigureS2A-PTBP1

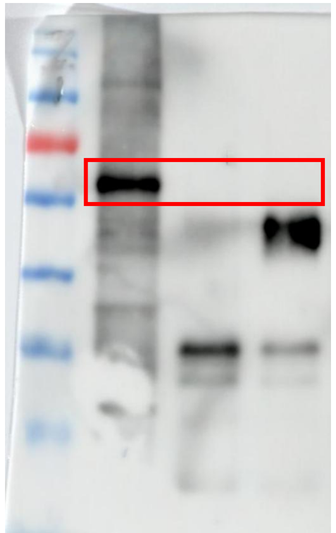

FigureS2A-FBL

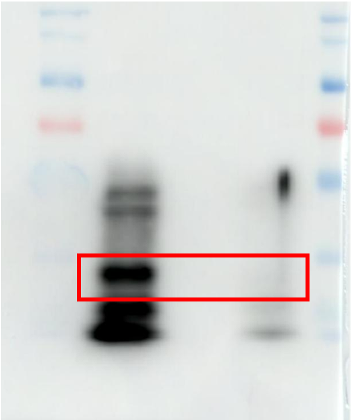

FigureS2A-HNRNPA0

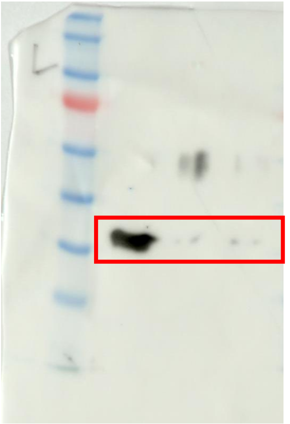

FigureS2A-SAFB

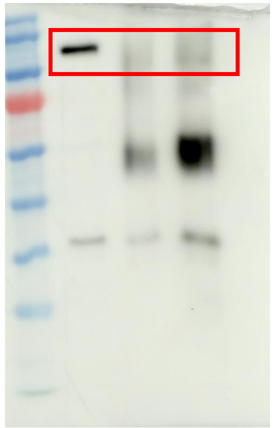

FigureS2B-USP5-450

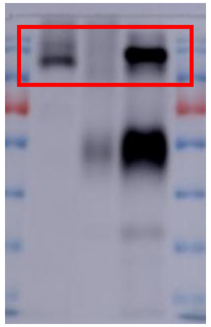

FigureS2C-IP:MYC-MYC

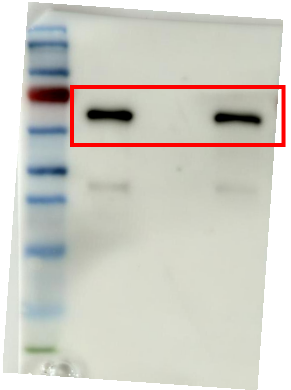

FigureS2C-IP:MYC-FLAG

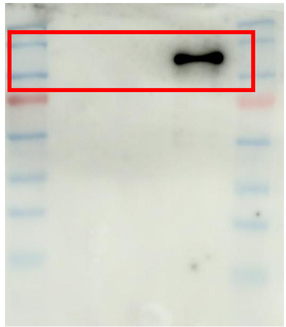

FigureS2C-IP:MYC-INPUT-MYC

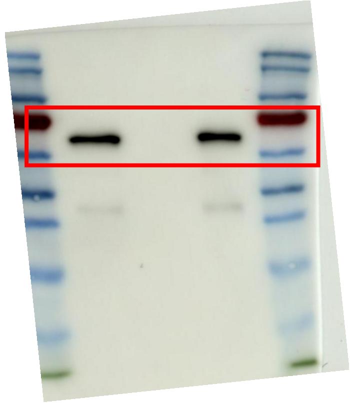

FigureS2C-IP:MYC-INPUT-FLAG

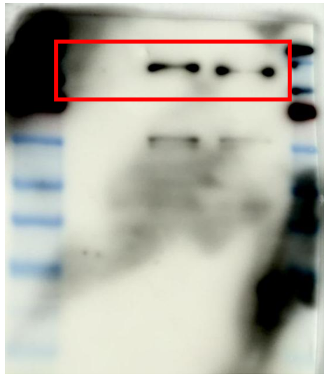

FigureS2C-IP:MYC-GAPDH

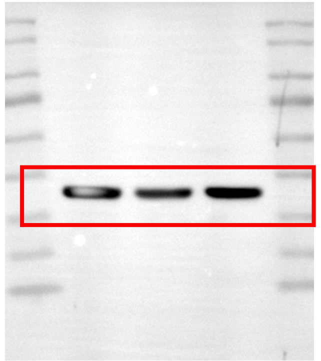

FigureS2F-USP5-KYSE150

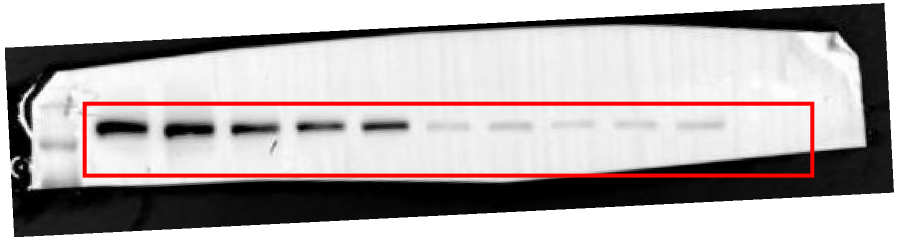

FigureS2F-IMPDH2-KYSE150

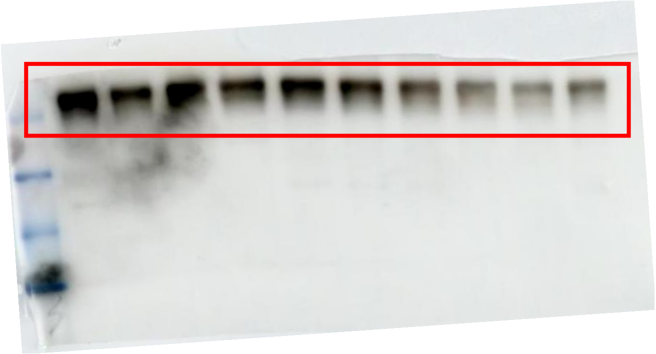

FigureS2F-GAPDH-KYSE150

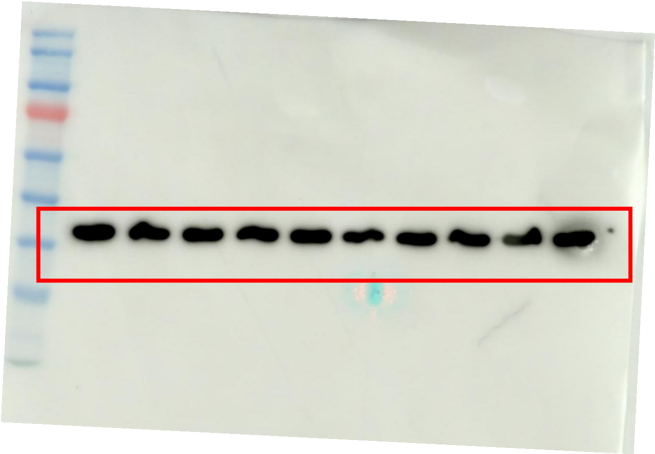

FigureS2G-MYC-HEK293

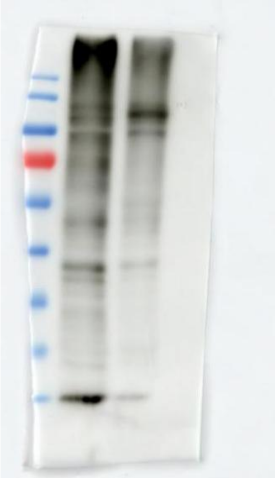

FigureS2G-INPUT-HA-HEK293

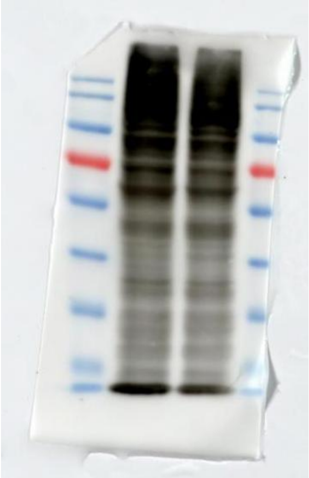

FigureS2G-USP5-HEK293

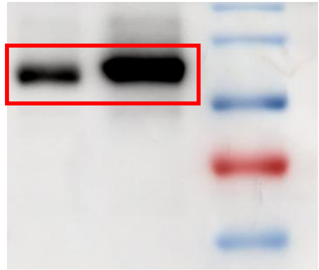

FigureS2G-GAPDH-HEK293

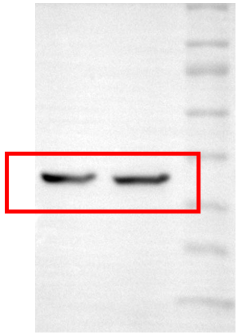

FigureS2H-KYSE70-Ub

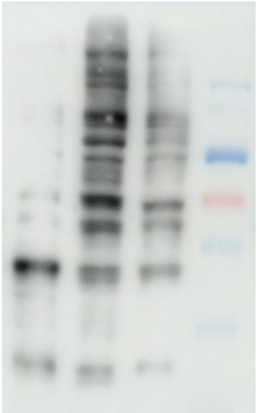

FigureS2H-KYSE70-HA

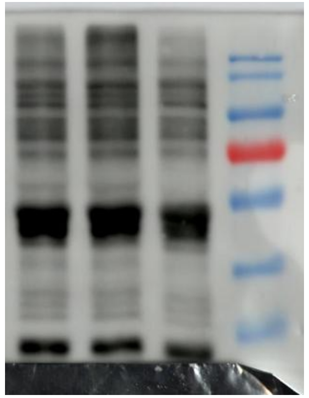

FigureS2H-KYSE70-USP5

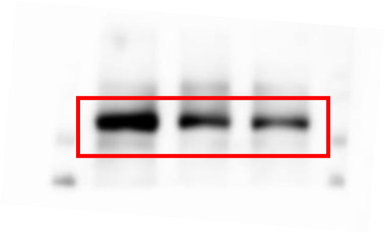

FigureS2H-KYSE70-GAPDH

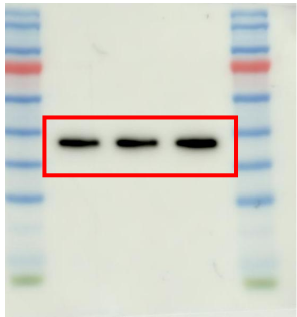

FigureS2H-Ub-KYSE150

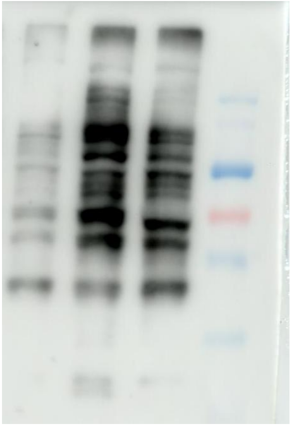

FigureS2H-HA-KYSE150

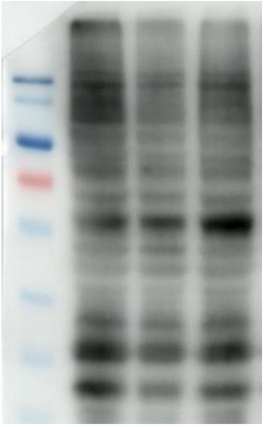

FigureS2H-USP5-KYSE150

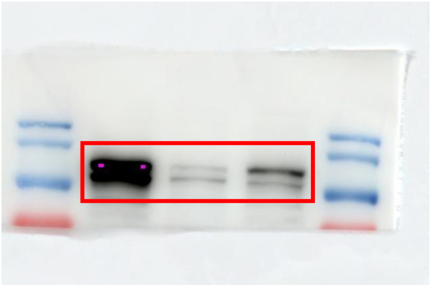

FigureS2H-GAPDH-KYSE150

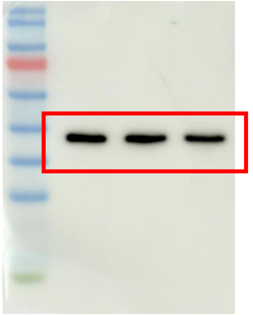

FigureS2I-HA

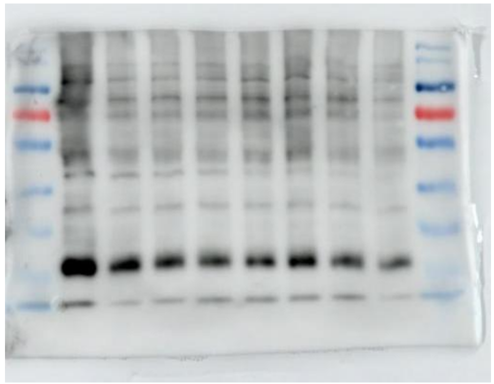

FigureS2I-HA

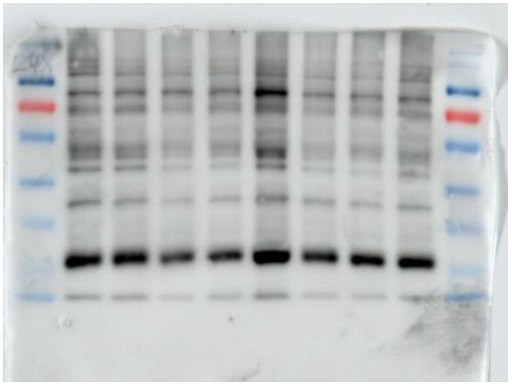

FigureS2I-INPUT

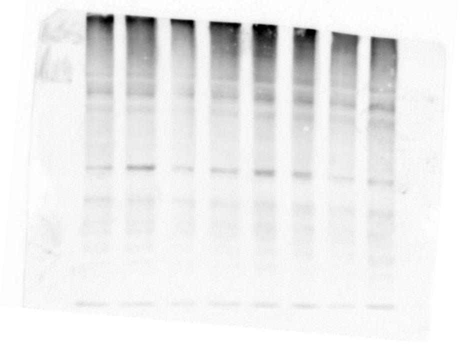

FigureS2I-INPUT

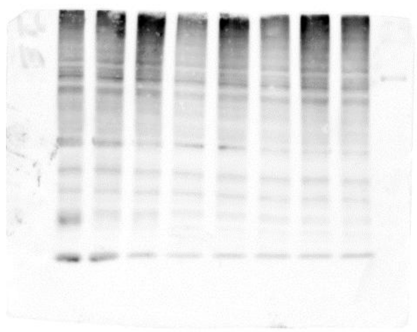

FigureS2I-USP5

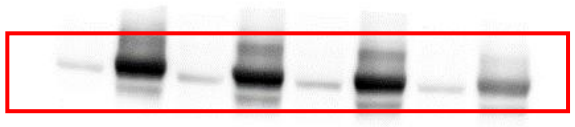

FigureS2I-USP5

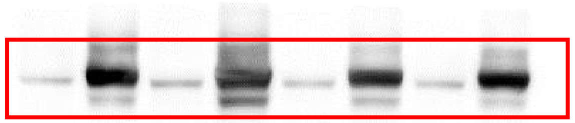

FigureS2I-GAPDH

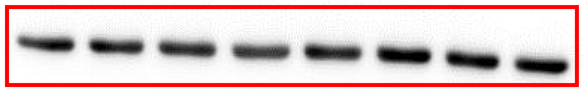

FigureS2I-GAPDH

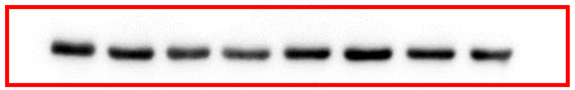

FigureS2J-Ub

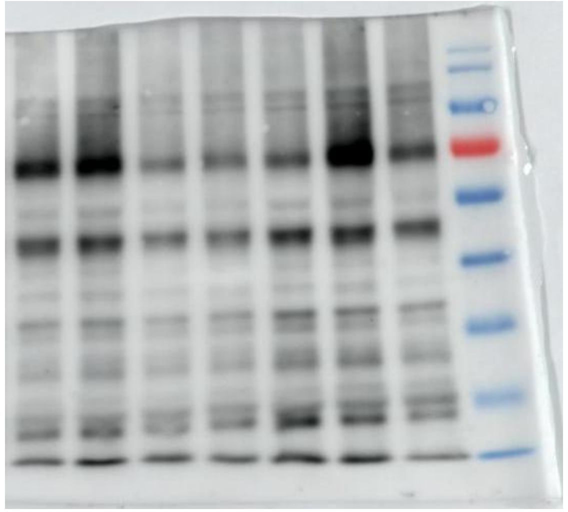

FigureS2J-INPUT-Ub

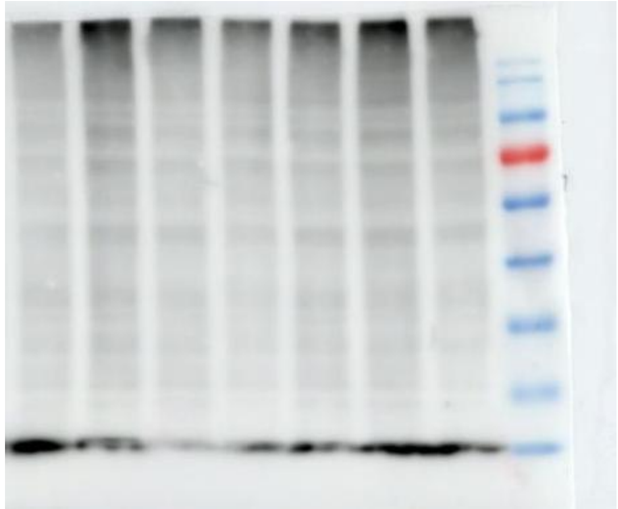

FigureS3C-IMPDH2

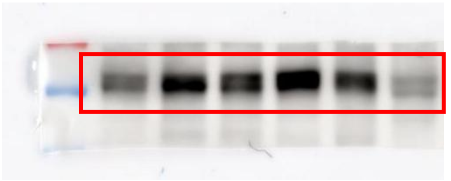

FigureS3C-IMPDH2

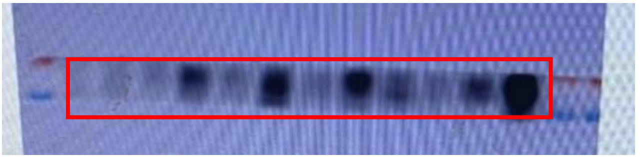

FigureS3C-GAPDH

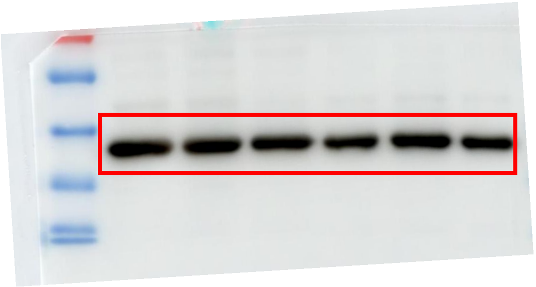

FigureS3C-GAPDH

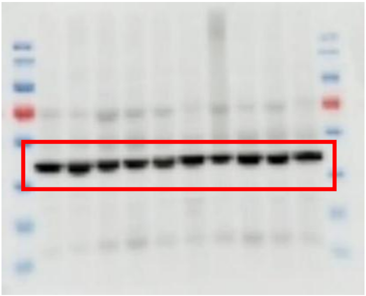

FigureS4A-IMPDH2-KYSE70

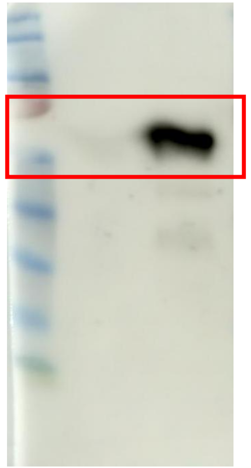

FigureS4A-GAPDH-KYSE70

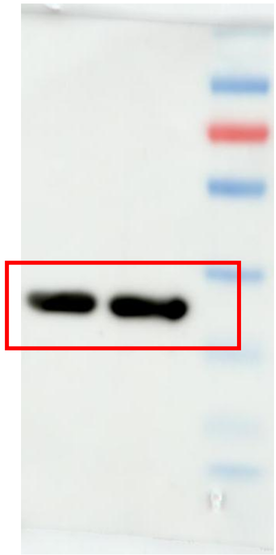

FigureS5D-SLC29A1

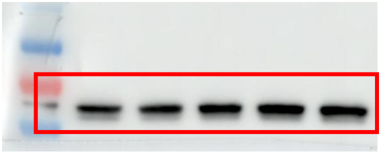

FigureS5D-GAPDH

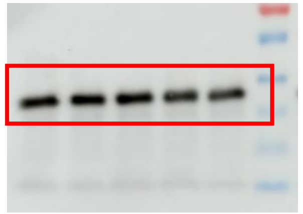

Supplement: Supplementary file 8 — original WB band [file 41419_2026_8683_MOESM8_ESM.pdf]
